# Fast effect size shrinkage software for beta-binomial models of allelic imbalance

**DOI:** 10.12688/f1000research.20916.2

**Published:** 2020-12-14

**Authors:** Joshua P. Zitovsky, Michael I. Love

**Affiliations:** 1 Department of Biostatistics, University of North Carolina at Chapel Hill, Chapel Hill, NC, 27516, USA; 2 Department of Genetics, University of North Carolina at Chapel Hill, Chapel Hill, NC, 27514, USA

**Keywords:** RNA-seq, Allelic imbalance, Allele-specific expression (ASE), Beta-binomial, Shrinkage estimation, Empirical Bayes, Bioconductor, Statistical software

## Abstract

Allelic imbalance occurs when the two alleles of a gene are differentially expressed within a diploid organism and can indicate important differences in cis-regulation and epigenetic state across the two chromosomes. Because of this, the ability to accurately quantify the proportion at which each allele of a gene is expressed is of great interest to researchers. This becomes challenging in the presence of small read counts and/or sample sizes, which can cause estimators for allelic expression proportions to have high variance. Investigators have traditionally dealt with this problem by filtering out genes with small counts and samples. However, this may inadvertently remove important genes that have truly large allelic imbalances. Another option is to use pseudocounts or Bayesian estimators to reduce the variance. To this end, we evaluated the accuracy of four different estimators, the latter two of which are Bayesian shrinkage estimators: maximum likelihood, adding a pseudocount to each allele, approximate posterior estimation of GLM coefficients (apeglm) and adaptive shrinkage (ash). We also wrote C++ code to quickly calculate ML and apeglm estimates and integrated it into the *apeglm* package. The four methods were evaluated on two simulations and one real data set. Apeglm consistently performed better than ML according to a variety of criteria, and generally outperformed use of pseudocounts as well. Ash also performed better than ML in one of the simulations, but in the other performance was more mixed. Finally, when compared to five other packages that also fit beta-binomial models, the *apeglm* package was substantially faster and more numerically reliable, making our package useful for quick and reliable analyses of allelic imbalance. *Apeglm* is available as an R/Bioconductor package at http://bioconductor.org/packages/apeglm.

## Introduction

Allelic imbalance (AI) occurs when the two alleles of a gene are expressed at different levels in a diploid organism, and its measurement is valuable in elucidating the factors that regulate the expression of genes. For example, for a diploid organism, the allele on one chromosome may have higher or lower expression levels compared to the allele on the other chromosome due to genetic variation in nearby non-coding regulatory sites, a process known as cis-regulation. AI in expression may also be associated with differential epigenetic state of the genomic region across the chromosomes. In some cases, differential allelic expression resulting from differential epigenetic state can be linked to the parent-of-origin of the alleles, a phenomenon known as genetic imprinting.

One challenge currently faced in allelic expression studies is that estimators for allelic expression proportions can be highly variable in the presence of low read counts and/or small sample sizes. Large estimates of allelic proportions in these cases often result from estimation error as opposed to true differences in allelic expression. Though small samples and low counts are a problem for RNA-seq data in general, they are especially problematic when dealing with allele-specific counts. When a subject is heterozygous at a particular SNP within an exon of a gene, RNA-seq reads that overlap the SNP allow for quantification of the levels of expression from either allele ^1^. Thus, allelic expression cannot be measured within a gene for subjects that are homozygous for that gene, and the number of samples with allele-specific counts for a gene can be much less than the number of samples in the study. Furthermore, alleles are often differentiated by a single SNP, and RNA-seq reads that do not overlap the SNP cannot be mapped to either allele. For these reasons, the proportion of RNA-seq reads that are allele-specific can be quite low, depending on read length and heterozygosity of the subjects. For instance, one study with 2x50 base pair (bp) paired-end reads and examining 30 million SNPs from 550 human subjects found that allele-specific counts made up 3.4% of RNA-seq reads ^2^. On the other hand, experiments making use of model organism crosses can maximize the number of RNA-seq reads overlapping heterozygous SNPs. For example, Raghupathy *et al*. ^3^ found in an RNA-seq dataset of a mouse F1 cross that 22% of uniquely mapping reads were allele-specific.

One traditional remedy investigators have used to deal with the challenges of high-variance estimators is to filter out genes that have low counts or small samples. While this does cause the resulting estimates to be more stable and thus representative of true allelic expression proportions, filtering may also remove genes that have true AI. One can think of this as akin to achieving higher specificity in detecting large true effect sizes at the cost of sensitivity. Furthermore, the cutoff used to determine what genes to filter out (i.e. how many counts a gene must have for it to not be removed) must be chosen per dataset by the analyst. Another traditional remedy has involved adding a pseudocount to each allele prior to estimation. As we will show, however, Bayesian shrinkage estimators offer advantages in moderating estimates.

A large number of Bayes estimators have already been developed for allelic expression studies. For instance, MMSEQ ^4^ uses a Gamma prior on allele-specific transcript abundance to provide AI estimates that are more accurate in the face of low coverage. Other methods that have used Bayesian approaches to test for AI include those by Leòn-Novelo *et al*. 2014 ^5^ and Skelly *et al*. ^6^ Leòn-Novelo *et al*. 2018 expanded on the work of Leòn-Novelo *et al*. 2014 and developed a method that can to estimate AI within groups as well as compare AI between groups ^7^. It uses Bayes estimators to shrink allelic proportions within groups toward 0.5, overdispersion toward a pre-specified prior mean, and the total counts of both alleles toward a pooled estimate. While more flexible than its predecessor, their method still does not allow for arbitrary design matrices (e.g. it cannot estimate the effects of continuous covariates on AI), and performance evaluations mainly focused on type I and type II error, not estimation accuracy. Since the original publication of our work, a Bayesian method for ASE has been developed which does allow for arbitrary design matrices ^8^. The method by Alvarez-Castro and Niemi 2019 models counts of each allele with over-dispersed Poisson regression models and places empirical Bayesian priors on both the regression and overdispersion parameters.

Though gene expression read counts are typically larger than allele-specific counts and can be measured for all subjects, the uncertainty of estimates in the presence of low counts and/or low sample sizes is still an issue. Thus, several shrinkage estimators for log fold changes in gene expression have also been developed which try to estimates that are only large due to the variance of the estimator and leave unchanged estimates that are likely to be large due to true expression changes ^9– 12, 13^. Many of these methods directly involve or can easily be applied to generalized linear models, which provide great flexibility in the kinds of study designs that can be treated and hypotheses that can be investigated. Though these methods were originally developed for improving accuracy and stability of log fold change estimates in gene expression, several can be directly applied or at least easily extended to estimating the effects of covariates on allelic expression proportions. For instance, Turro E, Astle WJ and Tavaré S ^13^ uses their method to assess imprinting by including haplotype information in the design matrix. Moreover, many of these methods have flexible generalized linear model specifications, and assessing allelic imbalance is as simple as changing the likelihood and link function appropriately (e.g. from a Poisson or negative binomial likelihood and log-link to a binomial or beta-binomial likelihood and logit link). We focus on the latter approach.

To this end, we look at four different estimation methods and their performance on data sets with small-to-moderate numbers of samples: maximum likelihood (ML), adding a pseudocount to each allele and sample, approximate posterior estimation of GLM coefficients (apeglm) ^12^ and adaptive shrinkage (ash) ^11^. ML estimators are based on estimating effects by modelling allele-specific counts with a beta-binomial GLM. Apeglm and ash are Bayesian shrinkage estimators which shrink likelihood-based estimates toward zero (ash can additionally handle non-likelihood-based estimates, such as quasi-likelihood estimates). Our results found that apeglm performed better than ML across a variety of metrics, making it robust and reliable when dealing with small sample sizes. Ash also performed better than ML in some metrics, though in other metrics results were more mixed. In addition to evaluating the performance of apeglm on allelic count data, we also introduced new source code for the apeglm package to improve computational performance for fitting beta-binomial GLMs and compared our improved package to other R packages that can also fit beta-binomial GLMs. As the apeglm package can calculate both ML and Bayesian shrinkage estimates, our improvements can be used even by those who wish not to use shrinkage estimators. Compared to other R packages, we show that apeglm with our improved code gives faster running times, greater scalability with the number of covariates, and better numerical reliability.

The methods and performance benchmarks we focus on here address issues stemming from low-count genes and small sample sizes. There are other important concerns in allele-specific analysis of short read RNA-seq datasets, such as reference allele bias, but we do not address such problems here and the methods discussed cannot directly account for them. Our simulation does not involve reference allele bias, and the RNA-seq study we examine took specific measures to avoid reference allele bias. For methods and analysis concerns involving reference allele bias, see Turro *et al.* ^4^ and Castel *et al.* ^1^

## Methods

### Estimation methods

We evaluated three estimation methods on their ability to estimate allelic expression proportions (or equivalently, the effects of covariates on allelic expression proportions): maximum likelihood (ML) estimation with the likelihood described below, approximate posterior estimation of GLM coefficients (apeglm) and adaptive shrinkage (ash). All analyses was done using R version 4.0.2 ^14^. The first two methods mentioned are implemented in the apeglm v.1.11.2 package, while the last is implemented in the ashr v.2.2.47 package. When using the ash function in the latter package, we set the method parameter equal to "shrink". While there are many Bayesian estimation methods that can be used to quantify allelic imbalance (AI), these allow for arbitrary design matrices. For instance, these methods can estimate differences in AI between groups while controlling for, or allowing interactions with, multiple additional variables, and can estimate the effects of continuous variables on AI.

For the *g*-th gene (1 ≤ *g* ≤ *G*), a beta-binomial GLM was fit to model allele-specific counts as follows. Let *Y _ig_* be the read counts of the first of the two alleles (which allele is designated as the first allele is arbitrary) for the *i*-th subject, 1 ≤ *i* ≤ *I*. Investigators may designate the first and second alleles of a gene as the paternal and maternal alleles or as the alternate and reference alleles, for example. It is assumed that $Y_{ig}\;\overset{ind}{∼}\;\text{BetaBin}(n_{ig},p_{ig},\phi_{g})$ where *n _ig_* is the equal to the total counts of both alleles for the *i*-th subject, *p _ig_* is the probability of reads belonging to the first allele of the *i*-th subject, and *ϕ _g_* is the overdispersion parameter. For the remainder of this paper, we will refer to the total allele-specific counts for both alleles of a particular gene and for a particular sample as the ‘total counts’ for that gene and sample. Furthermore, we will refer to the probability that a read for a particular gene belongs to a particular allele for a particular sample as the ‘allelic proportion’ for that particular allele and sample. The beta-binomial distribution models proportions that exhibit more variance than what would typically be observed under a binomial distribution (this additional variance is called the overdispersion), and is the typical distribution used for modeling allelic proportions. In this case the overdispersion *parameter ϕ* is inversely related to the actual overdispersion, and *ϕ* → ∞ implies variance no larger than what would be seen in a binomial distribution. *n*_1 *g*_, ..., *n _Ig_* are assumed to be fixed and known. As the beta-binomial probability mass function has multiple forms and parameterizations, we specify our parametrization as:

$$f(y;n,p,\phi )=\frac{\left(\begin{array}{c} n\\ y \end{array}\right)B(y+\phi p,n-y+\phi (1-p))}{B(\phi p,\phi (1-p))}$$

where *B* specifies the beta function. Furthermore, let **x** *_i_* be the *i*-th row of the design matrix **X** (matrix where columns are vectors of covariates of interest). Potential predictors include disease status for association studies, parent of origin for imprinting studies, and the presence of a SNP for eQTL linkage studies. We also assume that $p_{ig}={\left[1+\exp(-{\text{x}}_{i}^{T}\beta_{g})\right]}^{-1}$, or equivalently $\text{logit}(p_{ig})=\;{\text{x}}_{i}^{T}\beta_{g}\text{,}$ where ***β**_g_* = ( *β*_1 *g*_, ..., *β _Kg_*) *^T^* is a vector of coefficients representing the effect sizes for the predictors in the design matrix. For ML estimation, ***β**_g_* is estimated via ML. Constrained optimization is used for the nuisance parameter *ϕ _g_* with a minimum of 0.01 and a maximum of 5000 for the computational performance and numerical accuracy benchmarks and a minimum of 1 and a maximum of 500 for the estimation performance benchmarks. The user can specify the minimum and maximum as desired. The lower constraint is used for numerical stability as the evaluated probability mass function is degenerate for *ϕ _g_* = 0 and the upper constraint is used so that genes with no overdispersion do not have infinite estimated values of *ϕ _g_*, Details can be found in the ‘Estimating Overdispersion while Coefficients are Fixed’ section of the Supplementary Methods section ^15^. We found that using a range beyond a minimum of 1 and a maximum of 500 led to only very small, clinically meaningless differences in the coefficients, and we only went beyond this range to demonstrate our package’s potential numerical accuracy and computational robustness to larger overdispersion ranges. Standard errors and confidence intervals are calculated based on the asymptotic normal distribution of the ML estimators.

Apeglm shrinks the effects of one chosen covariate at a time, across all genes ^12^. It does this by assuming a zero-mode Cauchy prior distribution for the effects of one of the predictors. Due to its heavy tails, a Cauchy prior has a tendency to shrink truly large effect sizes less and in a differential gene expression context was shown to produce estimates with lower error and better ranking by size compared to a Normal prior ^12^. For estimating the effect of the *j*-th predictor in our model, where *j* ∈ {1, ..., *K*} is chosen by the user, we have:

$$\begin{array}{l} Y_{ig}|\beta_{g}\;\overset{ind}{∼}\;\text{BetaBin}(n_{ig},p_{ig},\phi_{g}),\;\text{for}\;\text{all}\;1\leq i\leq I,\;1\leq g\leq G\\ \;p_{ig}=\frac{1}{1+\exp(-x_{i}^{T}\beta_{g})},\;\text{for}\;\text{all}\;1\leq i\leq I,\;1\leq g\leq G\\ \;\beta_{jg}\;\overset{iid}{∼}\;\text{Cauchy}(0,\gamma_{j}),\;\text{for}\;\text{some}\;1\leq j\leq K\;\text{and}\;\text{all}\;1\leq g\leq G \end{array}$$

The scale parameter of the Cauchy prior, *γ _j_*, is estimated by pooling information across genes (see ‘Estimating the Scale of the Cauchy Prior’ section of the Supplementary Methods ^15^). Covariates other than the *j*-th covariate do not have their effect sizes shrunk, and instead we simply impose a wide and very weakly informative normal prior on their effect sizes (see ‘Estimating Coefficients while Overdispersion is Fixed’ section of the Supplementary Methods ^15^). Apeglm then provides Bayesian shrinkage estimates based on the mode of the resulting log-posterior of ***β**_g_*. Genes with lower expression, smaller numbers of heterozygous subjects and higher dispersion in allelic proportions will have flatter likelihoods, which will lead to the prior having more influence and shrinkage being greater. Furthermore, if the ML estimates are tightly clustered about zero, the estimated scale parameter of the Cauchy prior will be smaller. This will lead to more peakedness in the prior and also cause shrinkage to be greater. Posterior standard errors and credible intervals are calculated using a Laplace approximation to the posterior (we will use CIs to abbreviate both confidence intervals and credible intervals moving forward).

The original apeglm package estimated regression coefficients using C++ for negative binomial GLMs, while GLMs with other likelihoods, such as the beta-binomial, were fit completely in R. To improve scalability for large and/or high-dimensional data sets with beta-binomial GLMs, we wrote fast C++ code for calculating ML and apeglm shrinkage estimates of beta-binomial regression coefficients. We also changed the source code to speed up computation of the posterior standard errors (though such computations were still done in R) and prevent convergence issues. Details can be found in the ‘Estimating Coefficients while Overdispersion is Fixed’ section and the ‘Additional Technical Steps’ section of the Supplementary Methods ^15^. Finally, while we focus on optimizing performance and evaluating accuracy when a beta-binomial likelihood and Cauchy prior is used, we should note that the *apeglm* package can actually work with any custom likelihood function and any kind of generalized Student’s t prior (of which our default prior is a special case with zero mode and 1 degree of freedom).

Ash is a general Empirical Bayes shrinkage estimator for hypothesis testing and measuring uncertainty in a vector of effects of interest, such as a set of log fold changes in gene expression between biological conditions ^11^. Suppose again that one is interested in the effect sizes of the *j*-th predictor, ***β**_j._* = ( *β _j_*_1_, ..., *β _jG_*), where 1 *≤ j ≤ K*. Ash takes as input a vector of estimated effects ${\hat{\beta }}_{\mathrm{.j}}=({\hat{\beta }}_{j1},...,{\hat{\beta }}_{jG})$ (whether derived by ML estimation or some other method) and corresponding estimated standard errors ***σ**_βj._* = ( *σ _β j_*_1_ , ..., *σ _β jG_*). Here we take the estimated standard errors to be the true standard errors as suggested in the original methodology for ash, though the developers of ash have recently proposed an extension to their method that allows for random errors ^16^. For all 1 *≤ g ≤ G*, it is assumed that ${\hat{\beta }}_{jg}|\beta_{jg}∼N(\beta_{jg},\sigma_{\beta_{jg}})$ and that $\beta_{jg}\overset{iid}{∼}h_{j},$, where *h _j_* is some unimodal, zero-mode prior distribution. *h _j_* is estimated from the vector of estimates ***β**_j._* using mixtures of uniforms and a point-mass at zero, a choice guided by the fact that any unimodal distribution can be approximated as a mixture of uniforms with arbitrary accuracy ^11, 17, 18^. The posterior is $\beta_{jg}|{\hat{\beta }}_{jg}∼N(\beta_{jg},\sigma_{\beta jg})\times h_{j},$ and ash provides Empirical Bayes shrinkage estimates using the mean of the posterior. As ash uses the posterior mean, the point estimates will have minimum mean square error over the posterior. Genes with larger standard errors for their ML estimators will have a flatter likelihood that will be less impactful on the estimation. Thus, estimates for these genes will be shrunk more. Like apeglm, ash can only shrink estimates for one covariate at a time. Finally, it is worth noting that while the original paper and default *ashr* implementation use a normal approximation for the likelihood of estimates, and while we focus on this implementation here, the *ashr* package can alternatively work with a generalized Student’s t approximation, that is, ${\hat{\beta }}_{jg}|\beta_{jg}∼t_{df}(\beta_{jg},\sigma_{\beta_{jg}})$ (of which the Gaussian is a special case with infinite degrees of freedom). Posterior standard errors and CIs are calculated directly from the tail probabilities of the estimated posterior.

One of the main differences between apeglm and ash is that of assumptions. Apeglm assumes a fully-specified parametric model for the data likelihood and a specified generalized Student’s t prior and maximizes the resulting posterior from the full data. Ash only assumes the likelihood can be approximated with a normal distribution (or at least a generalized Student’s t distribution) and that the prior is zero-mode: By default, it uses a Gaussian approximation to the likelihood based on initial parameter estimates and associated standard errors, and the form of a zero-mode prior is determined empirically from the data and estimated in a nonparametric fashion via a mixture of uniforms. Though in large-sample settings the normal approximation will typically be close to the true likelihood (due to the Central Limit Theorem), there could be meaningful differences between the normal approximation and the true likelihood in small-sample settings. In these settings, if apeglm’s assumed likelihood is a good match, then it could benefit in avoiding approximation of the likelihood. However, if the specified likelihood is wrong, the likelihood approximation by ash could be more accurate. Moreover, as ash only assumes the prior is zero-mode and estimates it with a universal approximator, it is far more flexible and thus able to adapt to situations where a Cauchy prior (or generalized Student’s t prior more broadly) is inappropriate, such as if the true distribution of point estimates are non-symmetric or light-tailed. Furthermore, by only requiring a vector of arbitrary point estimates, ash can also work with estimates not derived by ML estimation, such as quasi-likelihood estimates.

In our software, we take several measures to preventing convergence issues and non-finite estimates even when the true ML estimate is non-finite ^15^. However, even with our imposed constraints, our optimization procedure can give ML estimates that are quite large (e.g. >7) if the genes have truly infinite ML estimates (e.g. one allele having zero count for all samples while the other allele has positive counts). In theory, these estimates could adversely affect estimation of the prior by ash and apeglm and lead to suboptimal shrinkage. To investigate the sensitivity of ash and apeglm to these estimates and possible solutions, we explored two possible remedies. First, we considered adding a pseudocount to each sample and for each allele prior to ML estimation. Second, we considered filtering out genes with Truly infinite ML estimates prior to ML estimation.

### Datasets and simulations

We compared the three estimation methods using the data set from the allelic expression study by Crowley *et al*. ^19, 20^ The study took mice from three divergent inbred strains (CAST/EiJ, PWK/PhJ and WSB/EiJ) and performed a diallel cross. The data set contains ASE counts for 72 mice and 23,297 genes in the resulting cross, with 12 mice of each possible parent combination (e.g. CAST/EiJ as mother and PWK/PhJ as father is one parent combination, and PWK/PhJ as mother and CAST/EiJ as father is another), and an equal number of males and females within each parent combination. Sequencing was performed with the Illumina HiSeq 2000 platform following the TruSeq RNA Sample Preparation v2 protocol to generate 100-bp paired-end reads. To assure that the mice all had the same alleles, we chose one genotype to focus on, namely the genotype resulting from the cross with CAST/EiJ and PWK/PhJ. The resulting data set, which we will refer to for the remainder of this paper as the ‘mouse data set’, had 24 mice, 12 of each sex and 12 of each parent of origin, and each mouse had nearly the same nuclear genetic composition as a result of the cross.

To evaluate the estimators on estimating effect sizes of predictors when the truth is known, we first fit an intercept-only beta-binomial model on each gene for the mouse data set. *ϕ* = [ *ϕ _g_*] is the vector of ML estimates of the overdispersion parameter from each model. 8 mice were then selected from the data set, 2 of each sex and parent of origin combination. Denote **N** *_I×G_* = [ *n _ig_*] as the matrix of total ASE counts for the 8 mice. Finally, a matrix of counts from one of the alleles **Y** *_I×G_* = [ *y _ig_*] was simulated for a sample size of 4 vs. 4, where *Y _ig_* was simulated from BetaBin( *n _ig_*, *p _ig_*, *ϕ _g_*), *logit*( *p _ig_*) = *β*_0 *g*_ + *β*_1 *g*_ *x*, *β*_0 *g*_ and *β*_1 *g*_ were both simulated from a standard normal distribution independently, and *x* splits the mice into two groups of size four ( *x* = 1 if a mouse is in the first group and 0 otherwise). Samples were drawn from the beta-binomial distribution using the emdbook v1.3.12 package ^21^. We refer to this simulation throughout the paper as the ‘normal simulation’, reflecting the distribution of the true effect sizes.

A second simulation was also performed that was similar in setup to the first, but with modifications to the distribution of *β*_1 *g*_ and *ϕ _g_*. In many studies, the effect sizes of a predictor will be zero for all but a handful of genes. Thus, *β*_1 *g*_ was simulated from *t*_3_ */*10 (a Student’s t-distribution with 3 degrees of freedom scaled by 1/10), which gave effects mostly close to zero, but with moderate and large effects occasionally appearing (Supplementary Figure 1 ^15^). Furthermore, the distribution of *ϕ _g_* from the mouse data appeared to be a mixture of two distributions: Genes without overdispersion had an obvious point mass at 500 with 70% proportion, and the remaining 30% of the genes could be modelled somewhat well by an exponential distribution with mean *μ* = 179 (Supplementary Figure 2 ^15^). To get more over-dispersed allele-specific counts, *ϕ _g_* was simulated from 0.5Exp( *μ* = 89) + 0.5(500), a mixture distribution where one component was exponential with a mean of 89 and had 50% proportion, and the other component was a point mass at 500 and had 50% proportion. We refer to this simulation throughout the paper as the ‘Student’s t simulation’, again reflecting the distribution of the true effect sizes. Note that these two simulations assume a data generating process, specifically the same data generating process as our assumed likelihood.

The estimators were then evaluated on real data with the focus on estimating mean, or gene-wide, AI. From the mouse data set, random samples of size 6 were drawn, and this process was repeated 10 times to reduce the impact of sampling variability. We will refer to these samples throughout the paper as the ‘random subsamples’. For each random subsample, the ML, apeglm and ash estimates of intercept-only models were calculated for the genes (where the intercept term was shrunk). Estimating the intercept in an intercept-only model for each gene is equivalent to estimating overall AI for each gene. As the truth is unknown for real data, and as we don’t have enough samples to estimate the truth with high accuracy in an independent held-out sample (there are only 24 mice in the mice dataset overall), we focus on more qualitative comparisons and metrics that don’t require the truth, such as the degree of shrinkage and CI width.

Finally, to demonstrate the flexibility afforded by apeglm, we fit a model to the entire mouse data set (all mice) with two binary variables as well as an interaction between them, and investigated the effects of ash and apeglm for shrinking the interaction term. Let *Y _ig_* denote the counts for the first allele for sample *i* and gene *g*, 1 ≤ *i* ≤ 24, 1 ≤ *g* ≤ *G*. For all genes and all samples in the mouse data set, we fit the model *Y _ig_* ~ BetaBin( *n _ig_*, *p _ig_*, *ϕ _g_*) where *n _ig_* is the total gene expression counts of sample *i*, gene *g*, *ϕ _g_* is unknown (estimated), *p _ig_* = *β*_0_ + *β*_1 *g*_ *SEX _i_* + *β*_2 *g*_ *POE _i_* + *β*_3 *g*_ *SEX _i_* × *POE*, *SEX _i_* = *I*(mouse *i* is female) determines the sex effect and *POE _i_* = *I*(mouse *i* has strain CAST/EiJ as mother) determines the parent-of-origin effect. The interaction effect *β*_3g_ was shrunk.

Additional simulations were conducted for evaluating computational performance of our improvements to apeglm, to see how well they would scale to larger and more complicated data sets. Allele-specific counts were simulated in a similar manner as the apeglm vignette ^22^. Briefly, we have **Y**_100×5000_ = [ *y _ig_*] as our simulated count matrix for one allele with associated total count matrix **N**_100 *×*5000_ = [ *n _ig_*] where rows are samples and columns are genes, *y _ig_ ~* BetaBin( *n _ig_*, *p _g_*, *ϕ _g_*), *ϕ _g_ ~ U* (0, 1000), *p _g_ ~ N* (.5, 0.5 ^2^), *n _ig_ ~* NB( *µ _g_*, 1/ *θ _g_*), and *θ _g_*, *_g_* are based on the airway data set by Himes *et al*. ^23^ To see how well our improvements scaled with increasing numbers of covariates, the data were split multiple times into differing numbers of groups of approximately equal size, where the number of groups ranged from 2 to 10. With K groups, the design matrix was **X**_100 *×K*_= [ **1 x**_1_ ... **x**_*K –*1_], where **x** *_j_* is an indicator variable for the ( *j* + 1)-th group, or a row vector whose *i*-th element is 1 if the *i*-th sample is in the ( *j* + 1)-th group and 0 otherwise. A simulation was also conducted to see how well apeglm would work with continuous predictors. This time, **Y** and **N** was kept the same, but with the design matrix **X**_100 *×*4_ = ( **1**, **x**_1_, **x**_2_, **x**_3_) = [ *x _ij_*], where **x**_1_ = (1, 0, 1..., 1, 0) *^T^* separates the samples into two equally sized groups and *x _i_*_2_, *x _i_*_3_ *~ N* (0, 1). **x**_1_ is the covariate whose effect size estimates are shrunk.

### Data processing

Genes where at least three samples did not have at least 10 counts were removed, which we considered minimal filtering that shouldn’t decrease statistical power. Genes without at least one count for both alleles across all individuals were removed. Genes with a marginally significant sex or parent effect were removed from the simulations and the real data analyses involving intercept-only models , so that all samples could be assumed independent and identically distributed for all genes.

To determine whether sex or parent effects were significant, beta-binomial GLMs were estimated for each gene by ML, with a design matrix that included a sex effect (an indicator that was 1 if male and 0 if female), a parent-of-origin effect (an indicator that was 1 if the mother was the CAST/EiJ strain and 0 if the father was the CAST/EiJ strain) and an interaction term. For each gene, if the p-value for the sex, parent-of-origin or interaction effect was less than 0.1, the effect was deemed marginally significant for that gene.

### Technical details of evaluations

For each gene, we define the shrinkage score as movement from the ML estimate to zero. We define a gene as (noticeably) shrunk if shrinkage exceeds 0.1, and substantially or most shrunk if shrinkage is greater than max $(1,|{\hat{\beta }}_{\text{ML}}|/4).$. For instance, if an apeglm estimate for a gene is 0.15 closer to zero than the ML estimate, then the shrinkage score is 0.15 and the gene is noticeably shrunk but not substantially shrunk by apeglm.

Concordance at the top (CAT) plots ^24^ were used to determine which estimation method could best find the most important genes (the genes with the largest effect size). For an estimation method, CAT takes the top genes according to the true ranking and compares it to the top genes according to the estimates, where the top genes are the genes with the largest true or estimated effect sizes in absolute value. For instance, a concordance at the top 10 of 90% means that the top 10 genes according to the estimation method and the top 10 genes according to the truth agree for 9 out of 10 genes.

For each of the design matrices posited in our computation simulations, computational performance of apeglm estimation was compared between the old and new apeglm code. From apeglm v1.11.2, we set the method parameter equal to “betabinCR" to run the new C++ code, and set the log.lik parameter equal to a beta-binomial log-likelihood function to run the old code from before our improvements were introduced (version 1.6.0 of the package). Details can be found in the vignette ^22^. Computational performance of ML estimation was also compared between our improved apeglm package and the following packages: aod v1.3.1 ^25^, VGAM v1.1.3 ^26^, aods3 v0.4.1.1 ^27^, gamlss v5.2 ^28^ and HRQoL v1.0 ^29^. Computational performance was evaluated using the microbenchmark v1.4.7 package ^30^ for estimation of a single gene (we used the *microbenchmark* function and set *times=20L*) and elapsed time for estimation of all 5000 genes, on a 2012 15-inch MacBook Pro with an Intel Core i7-3720QM processor.

### Determining the optimal filtering rule

In addition to comparing the three estimation methods described above, ML estimation paired with optimal filtering criteria was also assessed via CAT. CAT was chosen over other benchmark metrics, such as mean absolute error, as the different number of genes after filtering would make comparisons between filtered ML estimation and the three unfiltered methods biased. Furthermore, as we were primarily interested in whether a good filtering rule even existed, the true ranking of genes was used to determine the filtering rule. We looked at three rules: 1) removing genes where less than half the samples had a minimum total count threshold, 2) removing genes where less than all the samples had a minimum total count threshold, and 3) removing genes where the sum of total counts across samples was less than a certain threshold. For the remainder of the paper, we will refer to the sum of total counts across samples as the ‘summed counts’ of a gene. For each rule, various different thresholds were looked at: {0, 10, ..., 200} were potential thresholds for rule 1, {0, 10, ..., 100} were potential thresholds for rule 2, and {0, 50, ..., 1000} were potential thresholds for rule 3. For each rule and threshold, the ML estimates were calculated and concordance among the top 50, 100, 200, 300, 400 and 500 genes were averaged. We will refer to the rule and threshold that had the best concordance as the ‘optimal filtering rule’.

## Results

### Normal simulation

**Figure 1.**
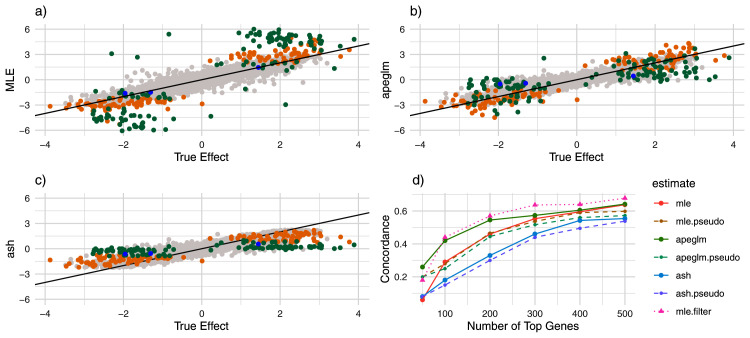
Estimate vs. truth and CAT Plots for normal simulation. **a**) Estimate vs. truth plot for ML estimation. Blue points represent genes substantially shrunk by apeglm only, orange points represent genes substantially shrunk by ash only and green points represent genes substantially shrunk by both ash and apeglm. **b**) estimate vs. truth plots for apeglm. **c**) estimate vs. truth plots for ash. **d**) CAT plot for the three methods with and without pseudocounts as well as for ML estimation after filtering. CAT: Concordance at the Top, ML estimation: Maximum Likelihood Estimation, apeglm: Approximate Posterior Estimation of Generalized Linear Model Coefficients, ash: Adaptive Shrinkage.

We began by looking at a simulation where allelic counts came from known beta-binomial distributions and effect sizes came from a standard normal distribution. In this simulation, apeglm and ML with pseudocounts successfully shrunk erroneously large estimates and had improved performance over maximum likelihood without pseudocounts. Ash had lower estimation error than ML, but estimation error was still higher than that of apeglm and ML with pseudocounts, and CAT performance was worse than ML (see Table 1 and Figure 1).

**Table 1.** Performance Metrics for Normal Simulation. ML: Maximum Likelihood, apeglm: Approximate Posterior Estimation of Generalized Linear Model Coefficients, ash: Adaptive Shrinkage.

Performance Metric	ML	Apeglm	Ash	ML+Pseudo	Apeglm+Pseudo	Ash+Pseudo
**MAE**	0.208	0.187	0.196	0.183	0.196	0.201
**MAE (apeglm-shrunk genes)**	0.626	0.501	0.552	0.487	0.561	0.577
**MAE (ash-shrunk genes)**	0.557	0.447	0.496	0.407	0.471	0.507
**MAE (counts<Q1)**	0.482	0.399	0.413	0.384	0.419	0.411
**MAE (counts>Q1, |MLE|>2)**	0.374	0.31	0.424	NA	NA	NA
**Coverage Probability for 95% CI**	0.949	0.94	0.924	0.935	0.911	0.9
**Average Interval Width for 95% CI**	1.109	0.862	0.813	0.795	0.751	0.736

Comparing apeglm and ash to ML first, we note that all three estimators have similar overall mean absolute error as many genes did not differ much between the methods ( Table 1). In exploring the behavior of shrinkage estimators, we were most interested in genes where shrinkage was high, and thus where estimates would be much closer to or much farther from the truth for one estimation method than for another. Thus, in addition to overall mean absolute error, we also calculated mean absolute error among genes that were noticeably shrunk by apeglm and genes that were noticeably shrunk by ash, to determine whether there was substantial improvement on average when apeglm or ash *did* noticeably shrink a gene. Shrinkage is defined as movement from ML to zero, and a gene is considered ‘(noticeably) shrunk’ by apeglm or ash if the apeglm or ash shrinkage exceeds 0.1 and ‘substantially shrunk’ if shrinkage exceeds max $(1,|{\hat{\beta }}_{\text{ML}}|/4).$ Among genes that were noticeably shrunk by apeglm, apeglm decreased the mean absolute error by 20%, and among genes that were shrunk by ash, ash decreased the mean absolute error by 20.8%. Moreover, among genes whose total counts were less than the 1 ^st^ quartile (and where shrinkage would be the most apparent), mean absolute error decreased by 17.2% and 14.3% for apeglm and ash respectively. We can also see that apeglm had lower mean absolute error than ash, both overall and across these three categories (apeglm-shrunk, ash-shrunk and low-count genes). As shrinkage can be considered a complex function of observed data statistics, stratifying by shrinkage does not use the true data-generating distribution and provides a fair comparison.

**Figure 2.**
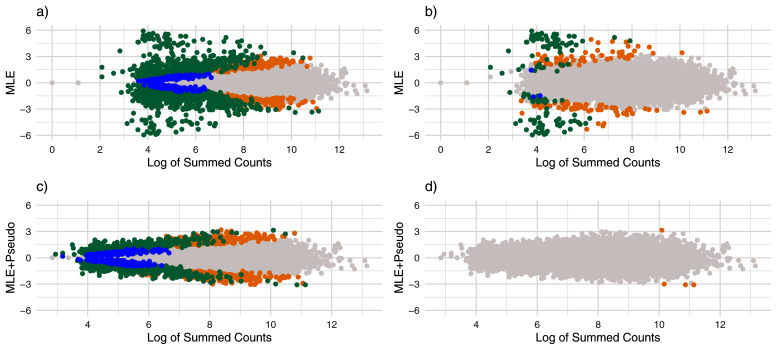
MA Plots for normal simulation. Estimates of effect size vs. log of summed counts. Each point represents a gene and the x-axis gives the logarithm of the gene’s summed counts. For the top plots ( **a** and **b**) the y-axis gives the ML estimates without using pseudocounts, and for the bottom plots ( **c** and **d**) the y-axis gives the ML estimates after adding pseudocounts. Points are colored by whether they were noticeably shrunk on the left ( **a** and **c**), and whether they were severely shrunk on the right ( **b** and **d**). Blue points represent genes noticeably or substantially shrunk by apeglm only, orange points represent genes noticeably or substantially shrunk by ash only and green points represent genes noticeably or substantially shrunk by both ash and apeglm.

From Figure 1a–c, it can be seen that apeglm shrunk most ML estimates that were inflated (i.e. much larger in magnitude than the corresponding true effect sizes), and mostly left truly large effects alone. Ash also shrunk ML estimates that were inflated, including some estimates less severely inflated that were missed by apeglm. However, ash also had a tendency to shrink more excessively, and that quite a few genes with truly large effects were shrunk to zero. This agrees with Supplementary Table 1 ^15^, which compares quantiles of shrinkage between apeglm and ash and illustrates a clear upward shift of shrinkage for ash. Moreover, from Figure 2a–b, we can see that both apeglm and ash exhibited more shrinkage for genes with low counts and severely shrunk genes with low counts and large estimates. However, ash also severely shrunk large ML estimates for genes with larger counts, even though these genes were more likely to have truly large effects. From Table 1, we see that among genes with high counts and large ML estimates, ash performed worse than ML estimation. All of this suggests that ash might be over-shrinking (that is, shrinking too much) for this data-generating distribution.

Adding a pseudocount to each sample and for each allele prior to ML estimation greatly improved accuracy of the ML estimates. Overall, the mean absolute error for adding pseudocounts followed by maximum likelihood was lower than ash and slightly lower than apeglm. However, adding pseudocounts prior to apeglm or ash actually made performance worse. From Figure 2c–d, it can be seen that adding pseudocounts alone lead to noticeably smaller MLEs, and this shrinkage was substantial for genes with low counts.

Apeglm greatly outperformed ML estimation in determining the set of genes with the largest effect sizes, where concordance at the top (CAT) was higher regardless of the number of genes being considered ( Figure 1d). Though adding pseudocounts improved CAT performance of the ML estimates, the improvement was modest and did not rival performance of the apeglm estimates. Ash performed worse than both apeglm and ML estimation, perhaps due in part to the potential over-shrinking discussed in the previous paragraph. Like for mean absolute error, pseudocounts improved CAT performance for ML estimation but made apeglm and ash CAT performance worse.

In addition to adding pseudocounts, we also attempted to filter out the 114 genes (out of about 10,000) with truly infinite ML estimates. The resulting changes did not alter conclusions: apeglm still had the best CAT performance, and ash still had the worst (Supplementary Figure 3) ^15^. Finally, we tried filtering out genes with small counts, with the cutoff based on that which improved CAT performance the most (dubbed ‘mle.filter’ in the CAT plot above). This led to the ML estimates having slightly better CAT performance than other methods on average. Thus, for this simulation, it was possible to beat other methods using filtering alone (by a small margin), provided we used knowledge of the underlying data-generating distribution to decide the filtering rule.

The coverage probability for intervals estimated by apeglm, ash and ML with pseudocounts were 1%, 2% and 1.5% lower than nominal, respectively, but average interval width was also 22.3%, 26.3% and 28.3% narrower than the likelihood-based intervals without pseudocounts ( Table 1). Adding pseudocounts prior to using apeglm and ash led to lower coverage and interval width was not that different from those obtained by using pseudocounts and maximum likelihood.

### Student’s t Simulation

**Figure 3.**
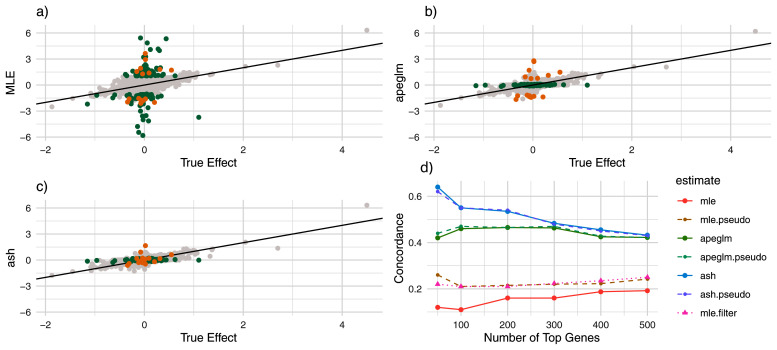
Estimate vs. truth and CAT Plots for Student’s t Simulation. **a**) estimate vs. truth plot for ML estimation. Orange points represent genes substantially shrunk by ash only and green points represent genes substantially shrunk by both ash and apeglm. All genes substantially shrunk by apeglm were shrunk by practically the same amount or more by ash. **b**) estimate vs. truth plots for apeglm. **c**) estimate vs. truth plots for ash. **d**) CAT plot for the three methods without and with pseudocounts. CAT: Concordance at the Top, ML: Maximum Likelihood, apeglm: Approximate Posterior Estimation of Generalized Linear Model Coefficients, ash: Adaptive Shrinkage.

We also investigated the performance of the estimators when most of the effect sizes were close to zero and overdispersion was large. Here both apeglm and ash gave marked improvement over the ML estimates, while the improvement from adding pseudocounts was only slight (see Table 2 and Figure 3).

**Table 2.** Performance metrics for Student’s t Simulation. MAE: Mean Absolute Error, MLE: Maximum Likelihood, apeglm: Approximate Posterior Estimation of Generalized Linear Model Coefficients, ash: Adaptive Shrinkage.

Performance Metric	ML	Apeglm	Ash	MLE+Pseudo	Apeglm+Pseudo	Ash+Pseudo
**MAE**	0.208	0.098	0.091	0.182	0.096	0.09
**MAE (apeglm-shrunk genes)**	0.375	0.127	0.114	0.318	0.125	0.113
**MAE (ash-shrunk genes)**	0.361	0.129	0.115	0.308	0.127	0.114
**MAE (counts<Q1)**	0.364	0.112	0.104	0.272	0.108	0.103
**Coverage probability for 95% CI**	0.921	0.938	0.942	0.924	0.936	0.942
**Average Interval Width for 95% CI**	0.975	0.462	0.471	0.864	0.454	0.454

Apeglm improved mean absolute error by 52.9% among all genes, and by 66.1% among noticeably shrunk genes specifically ( Table 2). Ash improved mean absolute error by 56.3% among all genes and by 68.1% among noticeably shrunk genes specifically. Adding pseudocounts slightly improved mean absolute error for the ML estimates, but estimation error was still not nearly as low as apeglm and ash, and again combining pseudocounts with ash and apeglm did not lead to better performance than using ash or apeglm without pseudocounts.

**Figure 4.**
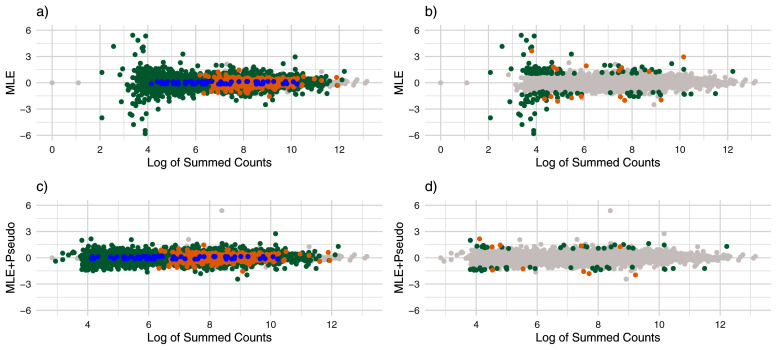
MA Plots for Student’s t Simulation. Estimates of effect size over log of summed counts for the Student’s t simulation. Each point represents a gene and the x-axis gives the logarithm of the gene’s summed counts. For the top plots ( **a** and **b**) the y-axis gives the ML estimates without using pseudocounts, and for the bottom plots ( **c** and **d**), the y-axis gives the ML estimates after adding pseudocounts. Points are colored by whether there were noticeably shrunk on the left ( **a** and **c**), and whether there were severely shrunk on the right ( **b** and **d**). Blue points represent genes noticeably or substantially shrunk by apeglm only, orange points represent genes noticeably or substantially shrunk by ash only and green points represent genes noticeably or substantially shrunk by both ash and apeglm.

Shrinkage patterns between ash and apeglm were much more similar here than in the normal simulation. Figure 3a–c show that both apeglm and ash shrunk inflated ML estimates similarly while leaving truly large effects mostly unchanged, though there a few inflated estimates that were missed by apeglm but shrunk by ash. Supplementary Table 2 ^15^ compares quantiles of shrinkage between apeglm and ash, and we can see that for this simulation the difference in shrinkage quantiles between apeglm and ash is quite small (though a paired Wilcoxon signed-rank test still concluded that ash had greater shrinkage on average with p<0.001). Furthermore, both ash and apeglm exhibited shrinkage for effects across the dynamic range of summed counts ( Figure 4a–b). This is not too surprising, as due to the increased overdispersion, there were more effects that were overestimated by ML, even among genes with large counts.

CAT performance was much better for apeglm and ash than for the ML estimates with and without pseudocounts, regardless of the number of top genes in question, with ash performing better than apeglm (particularly for the top 50 genes). Adding pseudocounts only slightly improved CAT performance for the ML estimates and did not improve performance for apeglm and ash. Moreover, filtering out genes with small counts (with the cutoff based on that which improved CAT performance the most) did not lead to nearly as good CAT performance for apeglm and ash. Thus, for this simulation, it was not possible to beat apeglm using filtering alone, even when using the true data-generating distribution to decide the filtering rule.

Both apeglm and ash had half the average interval width compared to ML despite also having higher coverage rates. Adding a pseudocount to each allele and sample followed by ML estimation did not lead to the same improvement in interval coverage or width and combining pseudocounts with apeglm or ash did not yield any improvement.

Filtering out genes with truly infinite ML estimates did not change prior estimation of apeglm or ash, as there were only 20 such genes for this simulation.

### Sampling from the mouse dataset

**Figure 5.**
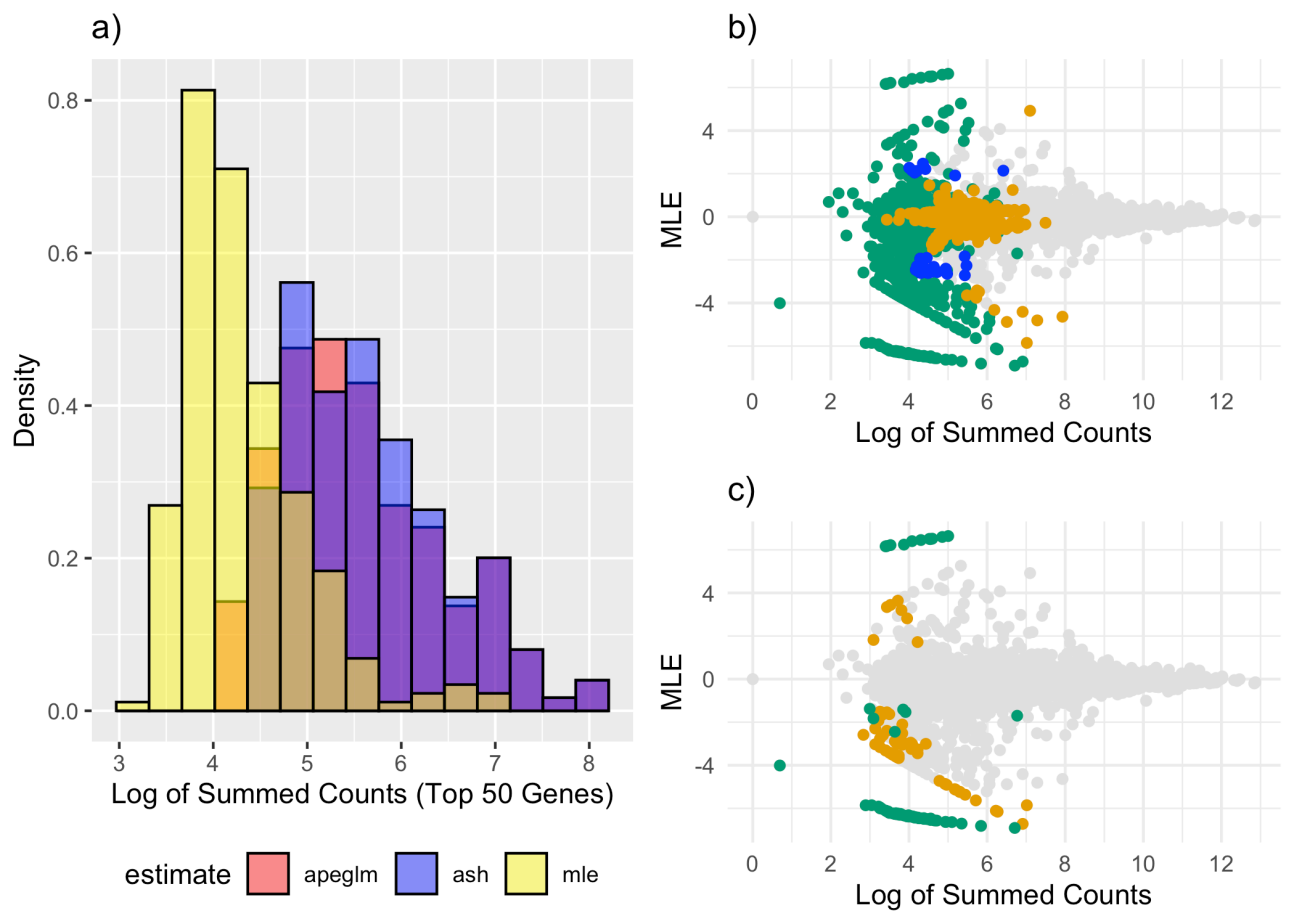
Distribution of Summed Counts for random subsamples. **a**) Overlapping histograms of log-summed counts for the top 50 genes according to ML (yellow), apeglm (red) and ash (blue), across all 10 subsamples. **b**) MA plot (ML estimates vs. log-summed counts) for one random subsample. Blue points represent genes noticeably shrunk by apeglm only, orange points represent genes noticeably shrunk by ash only and green points represent genes noticeably shrunk by both ash and apeglm. **c**) Same as ( **b**) except now points are only colored by whether there was substantial shrinkage, as opposed to whether there was noticeable shrinkage. ML: Maximum Likelihood, apeglm: Approximate Posterior Estimation of Generalized Linear Model Coefficients, ash: Adaptive Shrinkage.

To evaluate performance on real data, we first took 10 random subsamples of 6 mice from the mouse data set with replacement and calculated different evaluation metrics for each random subsample. These results are summarized in Table 3 and Figure 5.

**Table 3.** Summaries of Evaluation Metrics Across the Subsamples. For each gene, we define the “shrinkage score” as the movement from the ML estimate to zero. ML: Maximum Likelihood, apeglm: Approximate Posterior Estimation of Generalized Linear Model Coefficients, ash: Adaptive Shrinkage.

Evaluation Metric	ML	Apeglm	Ash
**75th Percentile of Shrinkage** **Scores**	NA	0.022	0.047
**90th Percentile of Shrinkage** **Scores**	NA	0.096	0.147
**97.5th Percentile of Shrinkage** **Scores**	NA	0.342	0.521
**99th Percentile of Shrinkage** **Scores**	NA	0.568	1.141
**Median Summed Counts of Top 50** **Genes**	63	222	248
**Average Interval Width for 95% CI**	0.567	0.43	0.395

Table 3 and Figure 5a are based on all subsamples. For example, with 10 subsamples and ~10,000 genes, the first row of Table 1 is the 75 ^th^ percentile of the ~100,000 shrinkage scores calculated across all 10 iterations, and each colored histogram in Figure 1a is based on the top 50 genes for each of 10 subsamples or 500 (possibly overlapping) summed counts overall. This was done to remove the effect of sampling variability on our results.

From Table 3, we see that percentiles of shrinkage scores were higher for ash than apeglm, particularly across the highest percentiles, indicating that ash was exhibiting more frequent and more severe shrinkage as in the normal simulation. It is also interesting to compare apeglm and ash in which genes they tend to shrink. For example, all of the genes shrunk by apeglm had low counts and/or very large MLEs, and this characterized many of the genes shrunk by ash as well ( Figure 5b–c). However, some of the genes shrunk by ash also had both larger counts and smaller ML estimates.

From Table 3 and Figure 5a, we can see that both apeglm and ash had higher counts among their top ranked genes than the top ranked genes by ML. For comparison, the 1 ^st^ quartile of summed counts of all genes was 507, and thus the distribution of counts for the genes ranked highest by apeglm and ash were more similar to the distribution of counts among all genes. Compared to ML intervals, intervals were 26.1% narrower for apeglm and 32.2% narrower for ash.

**Figure 6.**
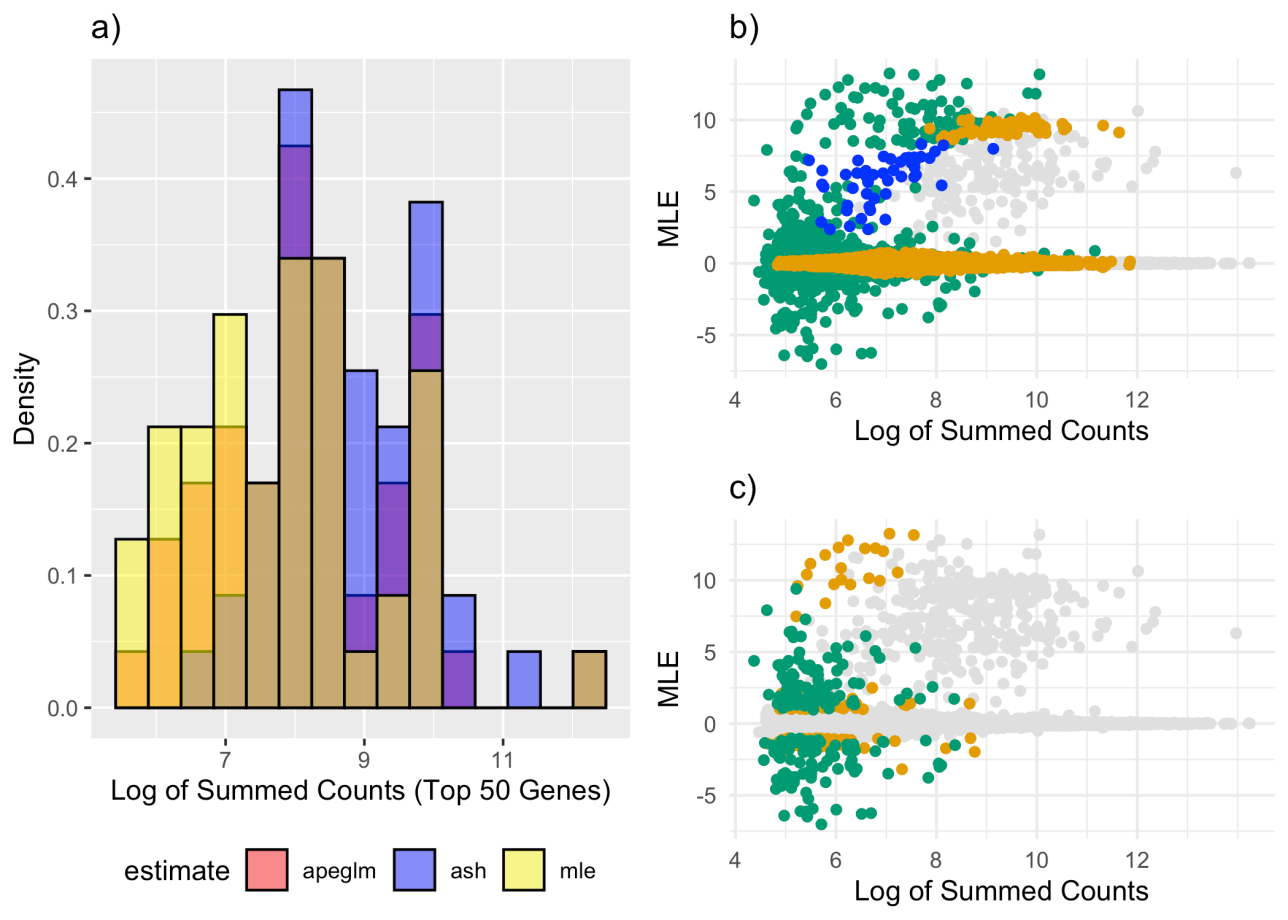
Distribution of Summed Counts for Interaction Model. **a)** Overlapping histograms of log-summed counts for the top 50 genes (genes with the largest interaction effect sizes) according to ML (yellow), apeglm (red) and ash (blue). **b)** MA plot (ML estimates vs. log-summed counts) for one random subsample. Blue points represent genes noticeably shrunk by apeglm only, orange points represent genes noticeably shrunk by ash only and green points represent genes noticeably shrunk by both ash and apeglm. **c)** Same as (b) except now points are only colored by whether there was substantial shrinkage, as opposed to whether there was noticeable shrinkage. ML: Maximum Likelihood, apeglm: Approximate Posterior Estimation of Generalized Linear Model Coefficients, ash: Adaptive Shrinkage.

We also fit a model with two binary variables and an interaction to all 24 mice, and used ash and apeglm to shrink the interaction term (see Table 4 and Figure 6). Unlike the real data intercept estimates and the estimates from the simulations, the distribution of ML estimates for the interaction effect had a positive mode and skew (sample skewness = 5.21). Apeglm assumes a symmetric distribution for the true effect sizes about zero and ash assumes at least a mode of zero, and it is not clear how much performance for apeglm and ash would degrade if these assumptions were violated. The ML estimates also had larger standard errors than in the simulations and real data intercept models, perhaps because there were three variables in our model instead of one and six mice per sex-POE group. Perhaps relatedly, ash estimates had a few notable differences than from previous analyses. For example, here average intercept width for ash was only 7.8% as large as that of likelihood-based intervals and only 12.8% as large as apeglm intervals. Filtering out infinite ML genes did not substantially change results (see Supplementary Table 3). 

**Table 4.** Summaries of Evaluation Metrics for the Interaction Model. ML: Maximum Likelihood, apeglm: Approximate Posterior Estimation of Generalized Linear Model Coefficients, ash: Adaptive Shrinkage.

Evaluation Metric	ML	Apeglm	Ash
**75th Percentile of Shrinkage** **Scores**	NA	0.023	0.188
**90th Percentile of Shrinkage** **Scores**	NA	0.134	0.427
**97.5th Percentile of Shrinkage** **Scores**	NA	0.697	1.213
**99th Percentile of Shrinkage** **Scores**	NA	1.693	2.334
**Median Summed Counts of Top 50** **Genes**	2582	3234	5616
**Average Interval Width for 95% CI**	1.275	0.795	0.111

### Computational performance of Apeglm

**Figure 7.**
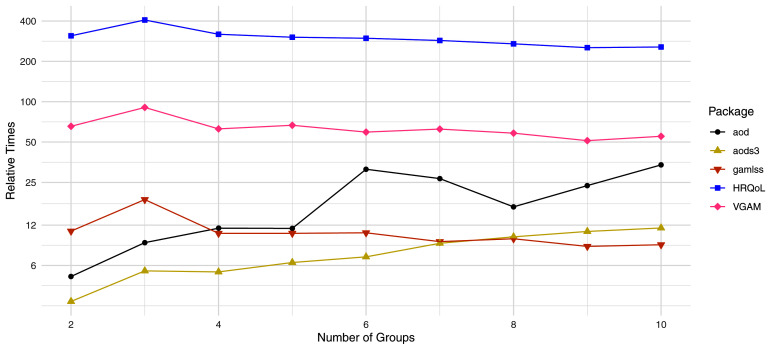
Comparisons in estimation time for one gene. Relative Times are defined as the fold changes in computation time relative to the *apeglm* package for the same number of groups. For instance, *aods3* takes about 6 times longer than *apeglm* to fit a beta-binomial GLM to one gene with two groups, and about 12 times longer than *apeglm* to fit such a model with three groups.

**Figure 8.**
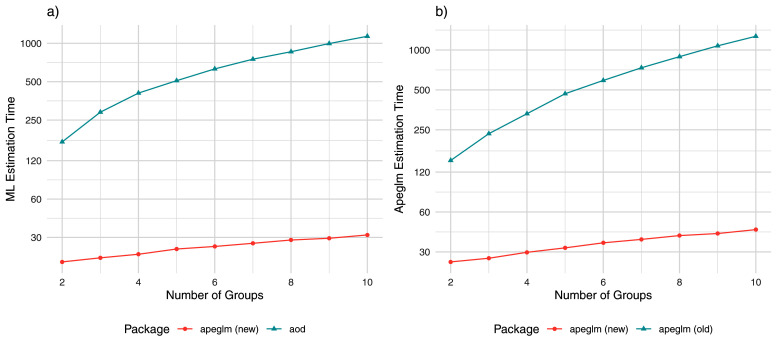
Comparisons in estimation time for all genes. **a**) computational time of ML estimation (in seconds) for the apeglm and aod packages by the number of groups (covariates). **b**) computational time of apeglm estimation for the new and old apeglm packages by number of groups (covariates).

To evaluate the computational performance of our package on larger datasets, we simulated allelic counts for 5000 genes and 100 samples, and randomly divided the samples into differing numbers of groups. apeglm with our improvements had very fast running times for both ML and apeglm estimation and scaled well with the number of covariates (see Figure 7 and Figure 8).

Estimation times per gene for ML estimation was substantially faster for apeglm than all other packages ( Figure 7). The next best package, aods3, took 3 to 12 times longer than apeglm and did not scale as well with the number of groups. Furthermore, the aods3. gamlss and HRQoL packages occasionally produced errors and could not fit beta-binomial models for all the simulated genes. Though our previous analysis showed that apeglm estimators often have higher accuracy than that of ML estimators, there are still reasons why one may prefer standard likelihood-based beta-binomial GLMs, such as if the sample size is large or if simplicity or unbiasedness is desired. Moreover, many shrinkage estimation packages like ash require a vector of initial ML estimates and standard errors, and one cannot use these methods without a ML-fitting package. It is also worth noting that apeglm estimation is practically as fast as ML estimation in the apeglm package, and thus for comparing apeglm estimation speed to ML estimation speed of the other packages, our package is still substantially faster.

For estimating all genes in the simulation via ML, apeglm took 24 seconds for two groups and added only 1–2 seconds of computational time for every group added ( Figure 8a). The next fastest package that could fit beta-binomial models for all the genes, aod, took seven times longer for two groups and grew 80 times as much for every group added. Comparisons in apeglm estimation between our improved apeglm package and the original package gave similar conclusions. Furthermore, unlike the new apeglm package, which grew roughly linearly with the number of groups in the range we assessed, the order of growth from the original package was not linear: the greater the number of groups already in the model, the greater the computational time increased for adding additional groups. At 10 groups, our improvements made apeglm 27 times faster than aod for ML estimation and 33 times faster than the old package for apeglm estimation. Our improvements also performed quite favorably when fitting beta-binomial models with two groups and two numerical controls. Elapsed time was 31 seconds for ML estimation and 43 seconds for apeglm estimation with the new apeglm package. In contrast, ML estimation took over nine minutes for aod and apeglm estimation took over seven minutes for the old apeglm package. Introducing multicollinearity into the design matrix did not substantially change computational performance for any package (results not shown).

In addition to looking at computational performance, we also compared the numerical accuracy and reliability of our package to the packages in Figure 7 using the same simulation. Gamlss, aods3 and HRQoL failed to converge and produced errors with relatively high frequency and estimates of aod and VGAM tended to have lower log-likelihoods than those of apeglm. Moreover, while aod did successfully converge for this computational simulation, it failed to converge (gave infinite estimates associated with log-likelihoods of negative infinity) when extreme overdispersion and smaller sample sizes were introduced into the simulation. On the other hand, our package converged in all evaluations, including across all simulations and real data analyses discussed here and in the Supplementary Methods ^15^. We imposed wide artificial caps on the sample-specific probabilities to prevent our package from producing errors and giving non-finite solutions even when the dataset of interest contains genes that exhibit all counts of zero for one allele across all samples, while positive counts for the other allele. For further details on numerical accuracy, see ‘Evaluating Numerical Accuracy’ section of the Supplementary Methods ^13^.

## Discussion

Here the performance of four estimators was compared across two simulations and one real dataset of allele specific expression in mice. The performance of the point estimates of apeglm was robust and consistent: across both simulations, apeglm had lower mean absolute error and higher concordance at the top than ML and had either the best or second-best estimation and CAT performance. Ash performed universally better than ML for the Student’s t simulation, but for the normal simulation its CAT performance was worse and its MAE was lower among genes with high counts. Conversely, use of pseudocounts and filtering performed comparatively similarly to apeglm in the normal simulation, but performed much worse than both apeglm and ash in the Student’s t simulation.

Apeglm and ash typically shrunk only low-count genes, as low-count genes tend to be those with the most uncertain and variable estimates. However, during a simulation where extreme overdispersion and heavy tails of the distribution of true effects were introduced, there were some large-count highly-variable genes that were shrunk by both methods as well, showing that ash and apeglm will shrink large-count genes if there is high uncertainty in the estimates. Ash consistently shrunk genes more than apeglm: the quantiles of shrinkage scores for ash were always higher than the corresponding quantiles for apeglm, and genes with high counts were more likely to be shrunk by ash.

No method gave confidence or credible intervals with the highest coverage rates for all scenarios. However, across both simulations, differences in coverage rates between the three methods were small, and coverage rates for apeglm credible intervals in particular were always very close to the interval that had the largest coverage. Furthermore, interval width for apeglm and ash were much smaller than that of ML. This suggests that interval estimates from apeglm and ash could have similar utility to and be advantageous over those by ML. For future research, it would be beneficial to evaluate the accuracy of Bayesian or frequentist hypotheses tests based on the estimates or posterior distribution of apeglm and ash using metrics such as type I and type II error. The method of Leòn-Novelo *et al*. 2018 ^7^ rejected hypotheses based on credible intervals of its posterior distribution, and if a similar step was taken for apeglm, its narrower intervals and robust coverage could potentially give more powerful hypothesis tests without suffering from inflated type I error.

Our changes to the apeglm package greatly improved computational performance for both ML and apeglm estimation of beta-binomial GLMs, particularly when larger numbers of covariates were involved. Among the R packages that we looked at which could fit beta-binomial models, the new apeglm package was the fastest for fitting many GLMs in sequence, e.g. across many genes or variant locations. It also had the best convergence in practice on datasets we evaluated: solutions had the highest likelihood on average, and it was one of the only packages that never produced errors or failed to converge, even in the face of extreme data dispersion and large ML estimates. The ML estimates were also indirectly capped to avoid non-finite solutions. For typical real datasets of reasonable size, it is common to have at least a few genes that exhibit counts of zero for one allele for all samples. Therefore, not only is apeglm substantially faster than the other packages, but it is also numerically accurate and reliable. Thus, the new apeglm package is useful for quick and reliable analyses of AI even for researchers who wish to only use likelihood-based estimators. Currently, only coefficient estimates are calculated in C++, and even better computational performance would be achieved if overdispersion and posterior standard error calculations were integrated into C++ as well. We are not aware of any other R packages made at the time of this article’s publication that utilize fast programming languages such as C or C++ to estimate numerous beta-binomial regression models based on large matrices of observed allelic counts. The most similar package we noted was fastglm ^31^, which fits individual quasi-binomial models in C++. While quasi-binomial models also estimate proportions and control for overdispersion, they do so in a different manner and with different assumptions.

Based on previous work, there are several ways in which the apeglm methodology could potentially be improved for allelic expression studies. For instance, while our extension of apeglm estimated overdispersion by ML estimation, the original methodology for apeglm as applied to negative binomial GLMs utilized Bayesian estimates for overdispersion as well as for regression coefficients. Introducing a prior for beta-binomial overdispersion that pools information across genes may lead to better estimation and inference of regression coefficients. We also assumed that the total allele-specific counts were fixed and known. Allowing such quantities to be random, as in the method by Leòn-Novelo *et al*. 2018, may lead to better inference as well. Adjusting for read mapping biases and ambiguities (Leòn-Novelo *et al*. 2014 ^5^; Leòn-Novelo *et al*. 2018 ^7^; Raghupathy *et al*. 2018 ^3^) could also lead to better estimates when such biases and quantification uncertainty are present. Lastly, though here we focused on beta-binomial GLMs, a wide variety of statistical models can be used for ASE, from quasi-binomial ^32^ to Poisson-lognormal models ^8^.

## Data availability

### Underlying data

Zenodo: RNA-seq Dataset from Crowley *et al*. 2015. http://doi.org/10.5281/zenodo.3404689 ^20^.

This project contains the following underlying data:

- fullGeccoRnaDump.csv

This file contains the Crowley *et al.* mouse dataset which was was obtained from http://csbio.unc.edu/gecco/data/fullGeccoRnaDump.csv.gz ^19, 33^. We uploaded the dataset to Zenodo on the authors’ behalf with their permission, due to the fact that the original dataset is not currently hosted in a stable repository.

The dataset from this repository is available under the terms of the Creative Commons Attribution 4.0 International license (CC-BY 4.0).

### Extended data

Zenodo: Supplementary Material for Zitovsky and Love 2019. http://www.doi.org/10.5281/zenodo.4033010 ^15^.

This project contains the following extended data:

- Supplementary Methods.pdf (Contains the mathematical and algorithmic details of how the apeglm package estimates beta-binomial coefficient effect sizes and reports results on its numerical accuracy)
- Supplementary Figures and Tables.pdf (Contains supplementary figures 1–3 and supplementary tables 1–3. These figures and tables were referenced and described in the main body of the article)

Data are available under the terms of the CC-BY 4.0 license.

## Software availability

Zenodo: Apeglm v1.11.2 Source Code. http://www.doi.org/10.5281/zenodo.4033035 ^34^. This repository contains the source code for the version of the apeglm package used in this paper.

The software from this repository is available under the terms of the GNU General Public License v3.0 (GPL-3).

Zenodo: Source Code for Zitovsky and Love 2019. http://www.doi.org/10.5281/zenodo.4033007 ^35^. This repository contains the R scripts used to run the analyses described in this article and generate all of its figures. All figures associated with this paper, including figures present in the main article and supplementary figures, were generated as separate .png or .eps files and can also be found in this repository. The R scripts can be found under the ‘Code’ folder while the figures can be found under the ‘Figures’ folder.

Material from this repository are available under the terms of the GPL-3 license.

apeglm is available as part of the Bioconductor project ^36^ at http://bioconductor.org/packages/apeglm. The vignette ^22^ and manual provide detailed information on how to use the package.
