# Supplementary material for: Fast effect size shrinkage software for beta-binomial models of allelic imbalance
Source: F1000Res. 2020 Dec 14;8:2024. Originally published 2019 Nov 28. [Version 2] doi: 10.12688/f1000research.20916.2 (PMC7974632; doi:10.12688/f1000research.20916.2)
Supplement: Peer review history

# Peer review: Reviewer response for version 2

**DOI:** 10.5256/f1000research.30569.r76123

**Published:** 2021-03-18

**Authors:** Matthew Stephens

**Affiliations:** 1 Department of Statistics, University of Chicago, Chicago, IL, USA; 2 Department of Human Genetics, University of Chicago, Chicago, IL, USA

The revision satisfactorily addresses my comments and I now approve this revised version of the article.

Is the rationale for developing the new method (or application) clearly explained?

Yes

Is the description of the method technically sound?

Yes

Are the conclusions about the method and its performance adequately supported by the findings presented in the article?

Partly

If any results are presented, are all the source data underlying the results available to ensure full reproducibility?

Yes

Are sufficient details provided to allow replication of the method development and its use by others?

Partly

Reviewer Expertise:

NA

I confirm that I have read this submission and believe that I have an appropriate level of expertise to confirm that it is of an acceptable scientific standard.

# Peer review: Reviewer response for version 1

**DOI:** 10.5256/f1000research.23018.r57280

**Published:** 2020-02-10

**Authors:** Ernest Turro

**Affiliations:** 1 Department of Hematology, University of Cambridge, Cambridge, UK; 2 MRC Biostatistics Unit, University of Cambridge, Cambridge, UK

This paper has two components:

 1) An advance in computational efficiency for estimating beta-binomial regression coefficients with shrinkage. The authors have produced a C++ implementation of the inference code previously written in R. Both versions of the code are implemented in the apeglm R package.

 2) An application of this new implementation of their method to the task of inferring allele-specific expression (ASE) and an assessment of its statistical performance in relation to two alternative approaches (ash and MLE).

 As the authors start the paper by discussing ASE, rather than computational inference for shrinkage models, it is not immediately apparent that the innovation presented in this paper is computational rather than statistical. Distinguishing these two components clearly would make it more readily apparent that the paper does not present a novel statistical method.

 The modelling of ASE has important facets that the authors do not discuss in the introduction (page 3) but which other (uncited) methods have addressed. For example, in a given sample, a gene may contain multiple heterozygous variants (potentially with uncertain phasing of alleles). Each heterozygous variant could overlap different sets of isoforms, each of which may have different levels of ASE. This phenomenon is modelled by the MMDIFF method (Turro *et al*, 2014, Bioinformatics ^1^), for example. The authors should acknowledge this (unmodelled) complication in ASE and explain how they summarise allele-specific count data across multiple variants (e.g., SNPs or indels, which are possibly unphased) within genes to obtain the count pairs modelled by the beta-binomial shrinkage estimators.

 The authors have performed several simulation studies and an analysis of a real ASE dataset. Both shrinkage estimators outperform MLE in the simulation studies. However, apeglm and MLE do approximately equally well in the real data set and both outperform ash by a significant margin. In addition, filtering of genes with low allele-specific read counts improves the MLE in the simulation studies but it does not do so in the real data analysis. This discordance demonstrates that the real data are very dissimilar from the simulated data. Although I don't think a major rewrite is warranted, if the authors could demarcate the computational advance (which can be demonstrated by simulation studies that are not representative of ASE, as the authors have done) from the specific application to ASE (using a real data set and perhaps a more faithful simulation study), the striking difference in performance shown in Figures 1-3 would be less incongruous.

 In the introduction, the inability of other methods to model the effects of continuous covariates or estimate differences in allelic imbalance between groups (this is not the case though, see MMDIFF) is highlighted and contrasted with the proposed method. However, the authors' own analysis of real data only uses an intercept model. It would be desirable to demonstrate the flexibility afforded by the proposed approach.

 In the assessment of statistical performance using the real data set, the MLEs obtained from the held-out data are treated as truth, even though earlier in the paper the authors demonstrate that MLEs have a particularly high mean absolute error. Presumably, this is the case (for genes with relatively low counts) even when the sample size is 18. The authors should consider alternative measures of performance that do not have this drawback.

 Minor comments:

- p3: "estimates for allelic expression proportions can be highly variable" - estimates are fixed, the authors should write "estimators".
- p3: a cancer dataset may not be the best choice of example to refer to the proportion of genes with allele-specific reads, due to the prevalence of somatic mutations.
- p3: when discussing filtering as a "remedy" perhaps explain that this achieves a boost in specificity at the cost of power.
- p3: "the most robust and reliable when dealing with small sample sizes" - this part of the sentence does not follow from the previous part, as there is no mention of ash's inadequacy.
- p3: "also introduced new source code" - it is not clear what the "also" refers to.
- p4: "the probability that counts for a particular gene belong to a particular allele" should be changed to "the probability that a read for a particular gene belongs to a particular allele" as the total "counts" will not be assigned to an allele as a block (the total counts derive from a heterogeneous mixture of reads from the two different alleles).
- p4: more information should be given about how the scale parameter of the Cauchy prior is "estimated by pooling information across genes".
- p4: the placement of the \cdot indexing the bold face beta is unusual, as the j subscript corresponds to the first rather than the second index.
- p9: rerunning the simulation study with 4 v 4 samples having run it with 5 v 5 samples seems unnecessary, as such a small change in sample size is unlikely to alter the conclusions.
- p9: "Figure 1d" should read "Figure 3d".

Is the rationale for developing the new method (or application) clearly explained?

Partly

Is the description of the method technically sound?

Yes

Are the conclusions about the method and its performance adequately supported by the findings presented in the article?

Partly

If any results are presented, are all the source data underlying the results available to ensure full reproducibility?

Yes

Are sufficient details provided to allow replication of the method development and its use by others?

Partly

Reviewer Expertise:

Biostatistics, genomics.

I confirm that I have read this submission and believe that I have an appropriate level of expertise to confirm that it is of an acceptable scientific standard, however I have significant reservations, as outlined above.

## References

[1] : Flexible analysis of RNA-seq data using mixed effects models. *Bioinformatics*.2014;30(2) : 10.1093/bioinformatics/btt624 180-8 10.1093/bioinformatics/btt624 24281695

# Response

**Published:** 2020-11-01

**Authors:** Josh Zitovsky

**Affiliations:** University of North Carolina at Chapel Hill, USA

This paper has two components:

 1) An advance in computational efficiency for estimating beta-binomial regression coefficients with shrinkage. The authors have produced a C++ implementation of the inference code previously written in R. Both versions of the code are implemented in the apeglm R package.

 2) An application of this new implementation of their method to the task of inferring allele-specific expression (ASE) and an assessment of its statistical performance in relation to two alternative approaches (ash and MLE).

 As the authors start the paper by discussing ASE, rather than computational inference for shrinkage models, it is not immediately apparent that the innovation presented in this paper is computational rather than statistical. Distinguishing these two components clearly would make it more readily apparent that the paper does not present a novel statistical method.

*We feel that the manuscript title referencing “software”, the abstract mentioning “we evaluated the accuracy of three different estimators” and “We also wrote C++ code to quickly calculate ... apeglm estimates”, the citation of the apeglm publication in the Introduction (“To this end, we look at three different estimation methods... approximate posterior estimation of GLM coefficients (apeglm) ^11^”), and the note about the software in the Introduction (“We also introduced new source code for the apeglm package”) make it clear that the apeglm shrinkage method is not proposed as novel in this manuscript.*

 The modelling of ASE has important facets that the authors do not discuss in the introduction (page 3) but which other (uncited) methods have addressed. For example, in a given sample, a gene may contain multiple heterozygous variants (potentially with uncertain phasing of alleles). Each heterozygous variant could overlap different sets of isoforms, each of which may have different levels of ASE. This phenomenon is modelled by the MMDIFF method (Turro *et al*, 2014, Bioinformatics ^1^), for example. The authors should acknowledge this (unmodelled) complication in ASE and explain how they summarise allele-specific count data across multiple variants (e.g., SNPs or indels, which are possibly unphased) within genes to obtain the count pairs modelled by the beta-binomial shrinkage estimators.

*We thank the reviewer for bringing up this concern. Here we have focused exclusively on observed allelic counts, ignoring uncertainty of reads that align to both alleles and aggregation of read information across SNPs within a gene. Such data could feasibly be acquired with longer reads that are approaching the transcript length, but in general we agree this as a limitation of our manuscript. We have now added the following to our manuscript to address this unmodelled complication:*

*“The methods and performance benchmarks we focus on here address issues stemming from low-count genes and small sample sizes. There are other important concerns in allele-specific analysis of short read RNA-seq datasets, such as reference allele bias, but we do not address such problems here and the methods discussed cannot directly account for them. Our simulation does not involve reference allele bias, and the RNA-seq study we examine took specific measures to avoid reference allele bias. For methods and analysis concerns involving reference allele bias, see Turro et. al. ^4^ and Castel et. al. ^1^."*

 The authors have performed several simulation studies and an analysis of a real ASE dataset. Both shrinkage estimators outperform MLE in the simulation studies. However, apeglm and MLE do approximately equally well in the real data set and both outperform ash by a significant margin. In addition, filtering of genes with low allele-specific read counts improves the MLE in the simulation studies but it does not do so in the real data analysis. This discordance demonstrates that the real data are very dissimilar from the simulated data. Although I don't think a major rewrite is warranted, if the authors could demarcate the computational advance (which can be demonstrated by simulation studies that are not representative of ASE, as the authors have done) from the specific application to ASE (using a real data set and perhaps a more faithful simulation study), the striking difference in performance shown in Figures 1-3 would be less incongruous.

*We thank the reviewer for pointing out that the simulation and real data results may have been seen as contradicting each other. Based on concerns voiced by other reviewers and our own investigations, we have determined that the issue is not in the simulations, but rather in the real data analyses. Specifically, when benchmarking our methods on the real data set, we had treated the ML estimates from a held-out set as the truth, but as the held-out set only contains 18 samples, the inherent instability and estimation variance present in ML estimators could still present an issue in the accuracy of these estimates. In other words, it may not be reasonable to expect that these ML estimates are close to the true effect sizes, and treating them as such could bias results in favor of ML estimates and against ash (as ash estimates are further from the MLE than apeglm on average). The real data analyses now have been changed to focus more on qualitative comparisons where the truth need not be known (e.g. extent of shrinkage, estimation variance, etc.), and we have largely left estimation accuracy assessments to the simulations. With these changes in place, the simulation and real data results are no longer incongruous.*

 In the introduction, the inability of other methods to model the effects of continuous covariates or estimate differences in allelic imbalance between groups (this is not the case though, see MMDIFF) is highlighted and contrasted with the proposed method. However, the authors' own analysis of real data only uses an intercept model. It would be desirable to demonstrate the flexibility afforded by the proposed approach.

*Thank you for bringing the Turro, Astle and Tavaré (2014) paper to our attention. We have added a mention of this paper in the Introduction as an example of a Bayesian method that can deal with allelic counts and arbitrary design matrices, and have removed the sentence that mentioned that methods do not exist to perform Bayesian analysis with arbitrary designs.*

*Moreover, we agree that it would have been useful to showcase our method on more complicated design matrices to demonstrate the flexibility of our method. To this extent, we have extended our analysis of real data to include an application of apeglm and ash to a model with two binary covariates and an interaction. The results are discussed in the last paragraph of the “Sampling from the mouse dataset” subsection of the “Results” section.*

 In the assessment of statistical performance using the real data set, the MLEs obtained from the held-out data are treated as truth, even though earlier in the paper the authors demonstrate that MLEs have a particularly high mean absolute error. Presumably, this is the case (for genes with relatively low counts) even when the sample size is 18. The authors should consider alternative measures of performance that do not have this drawback.

*We agree that treating the held-out MLEs as the truth is problematic and have changed the analyses of our real data set so that results do not depend on knowledge of the truth. See our previous response detailing this issue.*

 Minor comments:

- p3: "estimates for allelic expression proportions can be highly variable" - estimates are fixed, the authors should write "estimators".

           
*This typo has been corrected.*

- p3: a cancer dataset may not be the best choice of example to refer to the proportion of genes with allele-specific reads, due to the prevalence of somatic mutations.

*We now clarify that the TCGA dataset referenced here only used the normal breast tissue samples, not the tumor samples.*

- p3: when discussing filtering as a "remedy" perhaps explain that this achieves a boost in specificity at the cost of power.

           
*We have added this explanation as suggested. *

- p3: "the most robust and reliable when dealing with small sample sizes" - this part of the sentence does not follow from the previous part, as there is no mention of ash's inadequacy.

*We have changed this part of the sentence from “the most robust and reliable” to just “robust and reliable”.* 

- p3: "also introduced new source code" - it is not clear what the "also" refers to.

           
*We have changed this sentence to make it more clear.* 

- p4: "the probability that counts for a particular gene belong to a particular allele" should be changed to "the probability that a read for a particular gene belongs to a particular allele" as the total "counts" will not be assigned to an allele as a block (the total counts derive from a heterogeneous mixture of reads from the two different alleles).

           
*We have made the suggested change.*

- p4: more information should be given about how the scale parameter of the Cauchy prior is "estimated by pooling information across genes".

*We have added the mathematical details regarding how the scale parameter is estimated in the Supplementary Methods section.*

- p4: the placement of the \cdot indexing the bold face beta is unusual, as the j subscript corresponds to the first rather than the second index.

*We have made notational changes so that the \cdot appears after the j subscript and not before*

- p9: rerunning the simulation study with 4 v 4 samples having run it with 5 v 5 samples seems unnecessary, as such a small change in sample size is unlikely to alter the conclusions.

           
*Another reviewer made a similar comment, and so this result has been removed.*

- p9: "Figure 1d" should read "Figure 3d".

           
*The typo has been corrected.*

# Peer review: Reviewer response for version 1

**DOI:** 10.5256/f1000research.23018.r58251

**Published:** 2020-02-04

**Authors:** Jarad Niemi, Ignacio Alvarez-Castro

**Affiliations:** 1 Department of Statistics, Iowa State University, Ames, IA, USA; 2 University of the Republic, Montevideo, Uruguay

**Is the rationale for developing the new method (or application) clearly explained?**

 Yes.

- In our work a key issue is bias of allele reads toward a reference genome as explained in Sun and Hu (2014). ^1^ The authors should mention if this bias is relevant for the applications in this manuscript and, if yes, how the methods deal with the bias.
- The introduction argues against eliminating low count genes, yet the manuscript says "Genes where at least three samples did not have at least 10 counts were removed...Genes without at least one count for both alleles across all individuals were removed...Genes with a marginally significant sex or parent effect were removed." Why the contradiction?

**Is the description of the method technically sound?**

 No.

- While the writing is clear, we generally found the order of content confusing. For example, normal-based CI construction should be explained immediately after point estimation and before competing methods, simulation details, and method comparison metrics. We also found there was a lack of details, some of which was in the Supplementary Material but seemed like it should be included in the main manuscript.

In addition, we have outlined concerns below:

**Major concerns:**

- It isn't clear how MAE or CI coverage are calculated for the real data. For real data the truth is not known and therefore MAE and coverage cannot be calculated the way they can for the simulated data. Are you calculating MAE and coverage relative to the data? You comment "we are treating the MLE of the held-out set as the truth". Why? The simulation studies seemed to show this is a relatively poor estimate of the truth. 

**Minor concerns:**

- Please provide some statements for why a beta-binomial model is assumed as opposed to alternative model assumptions, e.g. binomial, normal, Poisson. 
- We assume you are assume conditional independence in your beta-binomial likelihood and in your Cauchy distribution for the regression coefficients. If so, this should be stated explicitly, e.g. using "ind" above the tilde.
- How often is \phi_g estimated to be 500? How important is the value 500? Is this user specifiable in the package?
- It is unclear what is meant by "standard error" in the statement "apeglm provides Bayesian shrinkage estimates based on the mode of the posterior as well as standard errors." Is this the posterior standard deviation? Is it the (asymptotic) standard deviation of the estimator? 
- The manuscript states "The scale parameter of the Cauchy prior, \gamma_j, is estimated by pooling information across genes". How exactly is this computed?
- It seems odd to have the Supplementary Material on a site other than F1000. We're disappointed that the Estimation Procedure in the Supplementary Material is not included in the main body of the manuscript as this seems to be key to the methodology. If not included in the main manuscript, perhaps more specific references, say to equation numbers, could be included in the main manuscript.
- We don't understand the statement "Like apeglm, ash can only shrink estimates for one covariate at a time." Isn't the assumed hierarchical distribution a joint hierarchical distribution, albeit assuming independence, for all regression coefficients? If so, then isn't it jointly shrinking all the estimates? Or is the procedure a step-wise procedure where MLEs are shrunk one-at-time?
- It is unclear why a Cauchy distribution is chosen. While a Cauchy distribution has the appealling property that it does not shrink large signals (very much), it generally does little shrinkage to small signals compared to alternative estimators, e.g. Bayesian LASSO (10.1198/016214508000000337,10.1093/biomet/asp047) ^2^^,^^3^, horseshoe (10.1093/biomet/asq017) ^4^, point-mass priors (10.1080/01621459.1993.10476353) ^5^. In our applications, the true distribution of these regression coefficients often has a large spike around 0 which would suggest using a distribution with more mass than a Cauchy near 0.
- The statement "where 1 <= j <= K is chosen by the user" is confusing. Does the user specify which predictors have a Cauchy distributions? What exactly is the user choosing?

**Are sufficient details provided to allow replication of the method development and its use by others?**

 Partly.

- One reason to provide code and data are to ensure ability to replicate even if the text is insufficient. So, ensuring the code is able to be run will provide sufficient details. 

**If any results are presented, are all the source data underlying the results available to ensure full reproducibility?**

 Yes.

- We also applaud the authors for making their code and data available.
  Reviewer 1 addressed this and we did not attempt to evaluate this further. 

**Are the conclusions about the method and its performance adequately supported by the findings presented in the article?**

 Partly.

 In the abstract, the article claims:

1. "Apeglm consistently performed better than ML[E] according to a variety of criteria, including mean absolute error (MAE) and concordance at the top (CAT)."
  Table 1 and 2 provide supporting evidence for the claim that apeglm has lower MAE than MLE for a variety of simulation scenarios.
  Figures 1d and 2d shows apeglm and ash having similar CAT and ahead of the non-filtered MLE approach. 
  It might be helpful to point out that ash, another shrinkage estimator, also consistently performs better than the MLE. 
2. "While ash had lower error and greater concordance than ML on the simulations, it also had a tendency to over-shrink large effects, and performed worse on the real data according to error and concordance."
  We guess Figures 1a-c and 2a-c as well as line 4 in Table 1 were the evidence for this comment, but we find these figures extremely hard to interpret. The comment in the text is that "some genes with estimates close to the truth were severely shrunk, and several genes with truly large effects were shrunk to zero.", but it isn't clear that this is undesirable. Just because the truth is non-zero doesn't mean that the data randomly generated from this truth should suggest a non-zero result.
  With this being said, we would not be surprised about ash shrinking large signals more than apeglm since the Cauchy distribution (used in apeglm) will shrink large signals less than a normal distribution (used in ash) will, but, as Reviewer 1 points out, there are differences in likelihood and estimation procedure between these two methods which make understanding why differences occur more difficult.
3. "2hen compared to five other packages that also fit beta-binomial models, the apeglm package was substantially faster, making our package useful for quick and reliable analyses of allelic imbalance."
  Figure 4 provides the computational cost comparison and seems to show that apeglm is faster than aod, aods3, gamlss, HRQoL, and VGAM under the tested scenario. An alternative version of this figure would provide the ratio of runtimes for these other methods compared to apeglm. While the current version allows for an understanding of the computation time involved, the main purpose of the figure is in comparison of times.
  It does seem a bit odd that the authors compared these packages for computation but not for accuracy. In addition, why is ash not included in this comparison?

**Other:**

**Minor issues:**

- Once you've defined an acronym, just use it, e.g. CAT.
- Be consistent with acronyms: choose ML or MLE and stick with it.
- Figure 5 seems unnecessary since an argument in this manuscript is to use "shrinkage" estimators rather than un-shrunk MLEs.
-  An updated reference for 29. Alvarez-Castro is 10.3934/mbe.2019389 ^6^
- The beta-binomial is a discrete random variable and thus it has a probability mass function rather than a probability density function.

Is the rationale for developing the new method (or application) clearly explained?

Yes

Is the description of the method technically sound?

No

Are the conclusions about the method and its performance adequately supported by the findings presented in the article?

Partly

If any results are presented, are all the source data underlying the results available to ensure full reproducibility?

Yes

Are sufficient details provided to allow replication of the method development and its use by others?

Partly

Reviewer Expertise:

Bayesian statistics

We confirm that we have read this submission and believe that we have an appropriate level of expertise to confirm that it is of an acceptable scientific standard, however we have significant reservations, as outlined above.

## References

[1] : Mapping of Expression Quantitative Trait Loci Using RNA-seq Data.2014; 10.1007/978-3-319-07212-8_8 145-168 10.1007/978-3-319-07212-8_8

[2] : The Bayesian Lasso. *Journal of the American Statistical Association*.2008;103(482) : 10.1198/016214508000000337 681-686 10.1198/016214508000000337

[3] : Bayesian lasso regression. *Biometrika*.2009;96(4) : 10.1093/biomet/asp047 835-845 10.1093/biomet/asp047

[4] : The horseshoe estimator for sparse signals. *Biometrika*.2010;97(2) : 10.1093/biomet/asq017 465-480 10.1093/biomet/asq017

[5] : Variable Selection via Gibbs Sampling. *Journal of the American Statistical Association*.1993;88(423) : 10.1080/01621459.1993.10476353 881-889 10.1080/01621459.1993.10476353

[6] : Fully Bayesian analysis of allele-specific RNA-seq data. *Math Biosci Eng*.2019;16(6) : 10.3934/mbe.2019389 7751-7770 10.3934/mbe.2019389 31698638

# Response

**Published:** 2020-11-01

**Authors:** Josh Zitovsky

**Affiliations:** University of North Carolina at Chapel Hill, USA

**Is the rationale for developing the new method (or application) clearly explained?**

 Yes.

- In our work a key issue is bias of allele reads toward a reference genome as explained in Sun and Hu (2014). 1 The authors should mention if this bias is relevant for the applications in this manuscript and, if yes, how the methods deal with the bias.

*Reference allele bias is indeed a potential problem when dealing with allelic counts from RNA-seq. However, the methods we benchmark in the manuscript cannot directly deal with such bias. Our simulation does not involve reference allele bias, and the RNA-seq study we examine took specific measures to avoid reference allele bias. We apologize for not clarifying this before and have added a paragraph at the end of the Introduction explaining these points. * 

- The introduction argues against eliminating low count genes, yet the manuscript says "Genes where at least three samples did not have at least 10 counts were removed...Genes without at least one count for both alleles across all individuals were removed...Genes with a marginally significant sex or parent effect were removed." Why the contradiction?

W *hen filtering is done to remove genes with a high variance estimated allelic ratio, it is usually done with a threshold greater than e.g. 10 total counts per gene / one count per allele. Increased filtering may result in a loss of statistical power, when the optimal filtering rule is not known. Our minimal filtering was performed such that the metrics (e.g. error and ranking concordance) represent features for which there is some minimally detectable signal across alleles.*

*Removing genes with a significant sex or parent effect was done for the purposes of performance analysis, as our analysis involved fitting intercept-only models. We did not want the extra variability induced from sex and/or parent effects in the set of genes used for evaluation.*

**Is the description of the method technically sound?**

 No.

- While the writing is clear, we generally found the order of content confusing. For example, normal-based CI construction should be explained immediately after point estimation and before competing methods, simulation details, and method comparison metrics. We also found there was a lack of details, some of which was in the Supplementary Material but seemed like it should be included in the main manuscript.

*We have moved the description of how the methods compute CIs as suggested. Moreover, we have added additional details about the estimation methods in both the main manuscript (under the “Estimation Methods” subsection of the “Methods” section) and the Supplementary Material. For example, in the main manuscript, we added more details regarding apeglm’s likelihood and prior, estimation of the overdispersion and qualitative differences between apeglm’s and ash’s methodologies. In the Supplemental material, we added more details regarding estimation of the overdispersion, estimation of the scale of the Cauchy prior and the numerical accuracy of our package.*

 In addition, we have outlined concerns below:

**Major concerns:**

- It isn't clear how MAE or CI coverage are calculated for the real data. For real data the truth is not known and therefore MAE and coverage cannot be calculated the way they can for the simulated data. Are you calculating MAE and coverage relative to the data? You comment "we are treating the MLE of the held-out set as the truth". Why? The simulation studies seemed to show this is a relatively poor estimate of the truth. 

           
*We thank the reviewer for noting this drawback in our initial submission. Initially, our choice to use the MLE of the held-out set as the truth came from the fact that the ML estimators are consistent and asymptotically efficiency estimators of the regression parameters, and thus if the held-out sets are sufficiently large, the ML estimates will be very close to the truth. However, the held-out set only consists of 18 samples, which in practice may be too small to be useful. We agree with your concerns that many of the same problems of ML estimators that we address in our manuscript, such as instability in the presence of low information, would still be present in the held-out sets. After thinking about this more and conducting additional analysis, we came to the conclusion that even when using as many as 24 samples, the ML estimates are not close enough to the truth for some genes and using them as the truth may bias results.*

*As a result, we have rewritten the real data analysis section to focus on qualitative assessments that do not require knowledge of the truth, such as differences in nature and extent of shrinkage between apeglm and ash and on estimation variance. Accuracy assessments have been largely left to simulations, where the true parameter values are known. Relatedly, we have changed the simulations so that the intercept is simulated from a standard normal distribution, as opposed to being drawn from ML estimates of intercept-only models fit to the genes of the real data set. The reason for this is similar: we have no reason to believe that the intercept ML estimates are close to the true intercepts, and upon investigation, we found that the distribution of ML estimates had several properties that would not realistically be demonstrated by a distribution of true effect sizes.*

**Minor concerns:**

- Please provide some statements for why a beta-binomial model is assumed as opposed to alternative model assumptions, e.g. binomial, normal, Poisson. 

*We have added a justification for choosing a beta-binomial distribution to model allelic counts in the second paragraph of the “Estimation Methods” subsection of the “Methods” section. *

- We assume you are assume conditional independence in your beta-binomial likelihood and in your Cauchy distribution for the regression coefficients. If so, this should be stated explicitly, e.g. using "ind" above the tilde.

*We have made the suggested changes to the notation so that the assumed conditional independence is clearer*

- How often is \phi_g estimated to be 500? How important is the value 500? Is this user specifiable in the package?

*It is difficult to give an exact frequency, as how often phi is estimated at 500 varies from dataset to dataset. The number of genes in a dataset where no or very little overdispersion is exhibited by the allelic proportions (conditional on the covariates) is roughly the number of times at which phi will be estimated at 500 for the dataset. As phi approaches infinity, the resulting regression parameter MLEs converge to the MLEs from a binomial distribution. We found that with phi=500, the ML estimates are already quite close to the ML estimates from a model with assumption of a binomial distribution, and setting the maximum above 500 led to only very small differences in the coefficients. However, the user can specify a different maximum (and minimum) than that used in this package as desired. Details have been added to the main manuscript and Supplemental Methods regarding our chosen minimum and maximum.*

- It is unclear what is meant by "standard error" in the statement "apeglm provides Bayesian shrinkage estimates based on the mode of the posterior as well as standard errors." Is this the posterior standard deviation? Is it the (asymptotic) standard deviation of the estimator? 

*It is the posterior standard deviation. We clarified this in the second version.*

- The manuscript states "The scale parameter of the Cauchy prior, \gamma_j, is estimated by pooling information across genes". How exactly is this computed?

*We have added this information in the Supplemental Material section*

- It seems odd to have the Supplementary Material on a site other than F1000. We're disappointed that the Estimation Procedure in the Supplementary Material is not included in the main body of the manuscript as this seems to be key to the methodology. If not included in the main manuscript, perhaps more specific references, say to equation numbers, could be included in the main manuscript.

*All references to the Supplemental Material have been made more specific, and are now references to the specific section of the Supplemental Material that is relevant.* 

- We don't understand the statement "Like apeglm, ash can only shrink estimates for one covariate at a time." Isn't the assumed hierarchical distribution a joint hierarchical distribution, albeit assuming independence, for all regression coefficients? If so, then isn't it jointly shrinking all the estimates? Or is the procedure a step-wise procedure where MLEs are shrunk one-at-time?

*We apologize if this was not clear in the first version of the manuscript and have added clarifications in the new version of the manuscript and Supplemental Material. In summary, apeglm for allelic counts assumes a Beta-binomial likelihood for all regression coefficients, but it only assumes a Cauchy prior for one regression coefficient at a time (more specifically, the regression coefficients for only one covariate, across all genes). Thus only one covariate is being “shrunk” at a time. If Bayesian shrinkage of two coefficients was desired (for example), you would have to run apeglm twice: the first time choosing one coefficient, and the second time choosing the other.*

- It is unclear why a Cauchy distribution is chosen. While a Cauchy distribution has the appealling property that it does not shrink large signals (very much), it generally does little shrinkage to small signals compared to alternative estimators, e.g. Bayesian LASSO (10.1198/016214508000000337,10.1093/biomet/asp047) 2, 3, horseshoe (10.1093/biomet/asq017) 4, point-mass priors (10.1080/01621459.1993.10476353) 5. In our applications, the true distribution of these regression coefficients often has a large spike around 0 which would suggest using a distribution with more mass than a Cauchy near 0.

*Our choice of a Cauchy prior was guided by the fact that a Cauchy prior tends to shrink large effect sizes less than other priors, and in a differential expression context was shown to produce estimates with lower error and better ranking be size than competing estimators (see reference 11). We agree that there are situations where a Cauchy prior would not be ideal, if sparsity of estimated coefficients (setting to exactly zero for certain genes) was desired for selection purposes. However apeglm follows and cites the ashr publication in providing the false sign rate (FSR) as a criterion for gene selection. A justification of our choice of a Cauchy prior and the flexibility of our software to handle other priors has also been added into the manuscript.  *

- The statement "where 1 <= j <= K is chosen by the user" is confusing. Does the user specify which predictors have a Cauchy distributions? What exactly is the user choosing?

*This is exactly right: The user is specifying which predictor (singular) is assumed to follow a Cauchy distribution for the purpose of shrinkage estimation. We have tried to make this clearer in the second version of the manuscript. See two responses above.*

**Are sufficient details provided to allow replication of the method development and its use by others?**

 Partly.

- One reason to provide code and data are to ensure ability to replicate even if the text is insufficient. So, ensuring the code is able to be run will provide sufficient details. 

*We apologize for the reproducibility issues present in the first part of the paper. A detailed explanation of the problems and our fixes was given in our responses to the first reviewer. We believe all previous issues have been fixed and the code should now run without problems (assuming all of the relevant packages are installed and the right package versions are being used).*

**If any results are presented, are all the source data underlying the results available to ensure full reproducibility?**

 Yes.

- We also applaud the authors for making their code and data available.
  Reviewer 1 addressed this and we did not attempt to evaluate this further. 

*Please see our response to your concern under “Are sufficient details provided to allow replication of the method development and its use by others?”.*

**Are the conclusions about the method and its performance adequately supported by the findings presented in the article?**

 Partly.

 In the abstract, the article claims:

1. "Apeglm consistently performed better than ML[E] according to a variety of criteria, including mean absolute error (MAE) and concordance at the top (CAT)."
  Table 1 and 2 provide supporting evidence for the claim that apeglm has lower MAE than MLE for a variety of simulation scenarios.
  Figures 1d and 2d shows apeglm and ash having similar CAT and ahead of the non-filtered MLE approach.
  It might be helpful to point out that ash, another shrinkage estimator, also consistently performs better than the MLE. 

*Due to changes in the simulations (see our response to your “Major Concern” under “Is the description of the method technically sound?”), ash no longer performs better than maximum likelihood universally, though in general it still performs better. The abstract has been changed to accommodate the different results. We believe that our new abstract provides a succinct yet comprehensive and accurate summary of the new results. *

1. "While ash had lower error and greater concordance than ML on the simulations, it also had a tendency to over-shrink large effects, and performed worse on the real data according to error and concordance."
  We guess Figures 1a-c and 2a-c as well as line 4 in Table 1 were the evidence for this comment, but we find these figures extremely hard to interpret. The comment in the text is that "some genes with estimates close to the truth were severely shrunk, and several genes with truly large effects were shrunk to zero.", but it isn't clear that this is undesirable. Just because the truth is non-zero doesn't mean that the data randomly generated from this truth should suggest a non-zero result.
  With this being said, we would not be surprised about ash shrinking large signals more than apeglm since the Cauchy distribution (used in apeglm) will shrink large signals less than a normal distribution (used in ash) will, but, as Reviewer 1 points out, there are differences in likelihood and estimation procedure between these two methods which make understanding why differences occur more difficult.

           
*Reviewer 1 voiced similar concerns, and you can see our detailed response to this concern in our responses to the first reviewer. To summarize, we have removed results of mean absolute error stratified by the true effect sizes. We also look more at subsets chosen based only on observed data (e.g. total allele counts and MLE size) to interpret results. We hope our new results are easier to interpret and our conclusions more convincing. *

1. "When compared to five other packages that also fit beta-binomial models, the apeglm package was substantially faster, making our package useful for quick and reliable analyses of allelic imbalance."
  Figure 4 provides the computational cost comparison and seems to show that apeglm is faster than aod, aods3, gamlss, HRQoL, and VGAM under the tested scenario. An alternative version of this figure would provide the ratio of runtimes for these other methods compared to apeglm. While the current version allows for an understanding of the computation time involved, the main purpose of the figure is in comparison of times.
  It does seem a bit odd that the authors compared these packages for computation but not for accuracy. In addition, why is ash not included in this comparison?

           
*We have changed the Figure as suggested to better illustrate relative performance of the other packages compared to apeglm. Moreover,  we have added comparisons of numerical accuracy to the main manuscript (last paragraph of “Computational performance of apeglm” subsection) and Supplemental Material. Our package is more numerically accurate and reliable than other packages compared. As to why ash is not included in the comparison, this is because ash requires a vector of initial parameter estimates and standard error estimates, and thus to use ash as we do in the manuscript, one has to perform ML estimation first, and then use ash to shrink the estimates. Comparing ash to apeglm or the ML-fitting packages would thus not be a same-to-same comparison.*

**Other:**

**Minor issues:**

- Once you've defined an acronym, just use it, e.g. CAT.

*We have made the suggested changes to the manuscript.*

- Be consistent with acronyms: choose ML or MLE and stick with it.

*We have made the suggested changes to the manuscript.*

- Figure 5 seems unnecessary since an argument in this manuscript is to use "shrinkage" estimators rather than un-shrunk MLEs.

*Though our previous analysis showed that apeglm has higher accuracy than ML estimators, there are still reasons why one would prefer likelihood-based beta-binomial GLMs, such as if the sample size is large or if simplicity or unbiasedness is desired. Moreover, many shrinkage estimation packages like ash require a vector of initial ML estimates and standard errors. Finally, apeglm estimation is almost as fast as ML estimation when using the new apeglm package, and thus Figure 5 would be practically the same if we were to compare other packages to apeglm estimation speed instead. We have added this clarification in the “Computational performance of Apeglm” subsection of the “Results” section. *

-  An updated reference for 29. Alvarez-Castro is 10.3934/mbe.2019389 6

*The reference has been updated.*

- The beta-binomial is a discrete random variable and thus it has a probability mass function rather than a probability density function.

*In the new manuscript, we refer to the probability function of the beta-binomial as its “probability mass function” as opposed to a “density function”*

# Peer review: Reviewer response for version 1

**DOI:** 10.5256/f1000research.23018.r57281

**Published:** 2019-12-17

**Authors:** Matthew Stephens

**Affiliations:** 1 Department of Statistics, University of Chicago, Chicago, IL, USA; 2 Department of Human Genetics, University of Chicago, Chicago, IL, USA

Summary:

 The paper presents new implementations of shrinkage methods for beta binomial models, implemented in the R software package apeglm. One potential application of these models is estimating allele-specific biases in various sequencing-based assays (and differences in bias between groups), and the paper focuses on this application.

 The performance of the shrinkage methods is assessed via simulation and real data analysis (using performance on hold-out data as a performance metric), and the shrinkage methods implemented here are found to be competitive with another shrinkage approach (adaptive shrinkage, ash), and consistently outperform the mle. The new implementations are also shown to be computationally faster than existing implementations (eg aod or the

 previous version of apeglm).

 The paper is generally well written, and carefully done, with some exceptions I note later. The new implementations seem likely to be useful in a range of applications. Certainly the use of shrinkage methods in these types of applications is to be encouraged, and I congratulate the authors for leading the way on this. I hope they will find my report helpful in revising their work.

 I was instructed "Please indicate clearly which points must be addressed to make the article scientifically sound." I believe points 2-4 below are most important to address to make the article scientifically sound.

**1. A note on differences between the shrinkage methods:**

 One thing that I felt was missing from the paper was a qualitative summary of how the two shrinkage methods used here differ from one another. Both are a form of Empirical Bayes shrinkage, but they use different prior families, different likelihoods, and different point estimate strategies: apeglm uses a Cauchy prior, with beta-binomial likelihood, and posterior mode point estimate; whereas ash uses a more flexible unimodal prior (which includes Cauchy as a special case), a normal approximation to the likelihood, and uses a posterior mean point estimate. So the trade-off here is that ash is using an approximate likelihood, but a more flexible prior and arguably a more principled point estimate (posterior mean is optimal under mean squared error).

 I think many readers might benefit from this "high-level" summary of the differences.

 Another important point, which will come up later, is that when using ash the user has a choice of how to make the normal approximation. Specifically ash requires the user to provide point estimates (beta-hat) and standard errors (s-hat), with the goal that beta-hat approx \sim N(beta, s-hat), where beta is true value that is being estimated.

 So there is not only one way to apply ash to a problem, but many different ways depending on the choice of point estimate beta-hat. The mle is one natural choice, but in this application there can be problems with infinite mles; see 2. below.

**2. On dealing with infinite mles:**

 To explain the issue with infinite mles, consider first a simple binomial experiment X \sim Bin(n,p) in which we observe X=0. Then the mle for p is 0, and the mle for theta:=log(p/(1-p)) is -Infinity. Similarly, if X=n the mle for theta is Infinity. Also, in both cases. the standard error for theta is infinite. The same issue arises in the more complex beta-binomial models considered here.

 Essentially if all the reads in an experiment show the same allele then the mle for the allelic bias parameter (on the logit scale) is +-Infinity. This could happen due to low coverage, but it could also happen at high coverage sites if the allelic bias is very strong.

 This issue appears to arise in the data analyses used to produce Figure 3 (I did not check whether it arises in the simulations). In Figure 3 there appear many mles (y axis) taking values near +-(5 to 6); however, my brief investigations of the data suggested that most of these likely correspond to genes where all the reads come from one allele, and so the mle is actually +-Infinity as above. (That these infinite mles are computed to be near +-6 is presumably due to an issue with the numerical maximization method used to compute the mle.)

 I suspect that the problems with ash observed in Fig 3 stem from this issue: the mle for these situations where all the reads come from one allele are very unstable, and have a very large standard error (technically infinite, although for numeric reasons finite values are used) and these large standard errors cause these mles to be shrunk excessively.

 A simple fix for this problem, and one I suggest the authors try, is to add a pseudo-count (say 1, or 0.5) to the counts for *each* allele in the data before computing "mles" and corresponding standard errors.

 Pseudo-counts are commonly used to improve stability of mles in this type of situation. Indeed, adding pseudo-counts can be viewed as a simple kind of shrinkage method, so it seems reasonable to compare the more sophisticated EB methods with the simple pseudo-count method. For most genes the point estimates and standard errors will be very little affected by the addition of a small pseudo-count; but for the problematic genes with infinite mle the pseudo-count will stabilize the point estimate and reduce the standard error. I suspect entering the stabilized estimates + standard errors into ash  will greatly reduce the problems observed with use of the mles in Figure 3.

 (Incidentally, Xing, Carbonetto and Stephens arXiv:1605.07787 ^1^ encounter a closely-related issue when using ash to smooth Poisson data; they solved this using a slightly different approach that is conceptually similar to adding a pseudo-count.)

**3. Subsetting results based on shrinkage amounts and "true" values:**

 In several places the paper reports error measures on subsets of the results. For example, in Table 1 lines 2-4 involve subsets of results chosen based on the true effect size or shrinkage amount (which depends on the true effect). Although tempting, this type of result is hard to interpret. For example, even the optimal shrinkage rule (i.e. the one that uses the correct prior, likelihood and loss function) may not perform uniformly better than the mle on subsets that are chosen in this way. Thus the sentence on p7 ("For instance, among genes with effect sizes greater than two...") may also be true for the optimal shrinkage rule, and so does not constitute direct evidence for "overshrinkage". (I agree there is overshrinkage, but this is not the right way to show it). Comparisons like p9 ("Among genes that were shrunk..."), which stratify by the amount of shrinkage, have the same problem because the amount of shrinkage depends on the true value and not only on the observed value.

 It is much cleaner and easier to interpret results if they are subsetted based on the *observed* effect (mle), rather than the true effect. This is because the optimal shrinkage rule is still optimal for *any subset chosen based only on the observed data*. (For this reason you could also subset based on other features of the observed data, like total allele count.) For example, if a method is worse than the mle for the subset of results where the mle is >4 then this is indeed evidence of a problem of some kind.

**4. Computation: speed vs accuracy:**

 When comparing with other methods/implementations there should be some assessment not only of speed, but of

 accuracy of the different implementations (meaning the accuracy with which they optimize the log-likelihood, rather than the accuracy of the point estimates). Fast answers are easy if you do not care about accuracy....

 E.g. I suggest boxplots of loglik(method) - loglik(apeglm-new) for each method, to show that the apeglm-new solution is consistently as high in log-likelihood as other methods (or nearly so). Are there convergence criteria decisions to be made that might affect the trade-off between speed and accuracy?

**5. Reproducibility:**

 I congratulate the authors on making all their code and data available. After a few tweaks to the code I was able to run the code used to produce Figures 1-3. However, my version of Fig 3 looked different from the one in the paper -

 my figure had different colors and some points seemed to be missing on my figure. I do not know the reasons for this.

 Reproducibility would have been made easier by avoiding the use of absolute file paths. I also suggest not defining functions that operate on global variables (e.g. subsetCalculations = function(sub){..,}) since they are more likely to lead to reproducibility problems.

 I was unable to run the code to perform the computation time comparisons (Figure 4), since it errored out. Again I do not know the reason, but it could be due to differences in the package versions I used compared with the authors. I did not have time to troubleshoot this.

**6. Miscellaneous other comments:**

 For Table 3, I think it should be noted that the coverage probability is expected to be <0.95 because you are looking at how often the interval covers the *estimate* in the larger dataset, and not the *true* value. This makes it a hard to compare the methods here because it isn't clear what the right coverage is.

 p12: "ash would most likely perform best in a situation where most effects were small". I don't see any evidence for this here (e.g. in the normal simulation ash performs fine) and indeed no reason to expect it to be true a priori. I think this statement should be removed.

**7. Minor comments:**

- p3: "When a subject is heterozygous for a gene at a particular SNP"; this wording seemed awkward to me.
- p3: "... making it the most robust and reliable when dealing with small sample sizes"; this conclusion ("making it") seemed not to follow directly from the first part of the sentence.
- p4: "Apeglm shrinks the effect of one predictor at a time": I think this sentence might work better at the start of the paragraph, before specifying the prior used.
- p5: "guided by the author's claim": this is not just a claim, it is a theorem dating back to the 1950s (see original paper for citations).
- p5: diallel typo?
- p5: use of beta for the mean of the exponential distribution is confusing as beta is already used elsewhere.
- p9: "We also conducted..." This did not seem worth reporting to me. The difference in sample size (5 vs 5 instead of 4 vs 4) is too small to expect that the results would be very different.
- p9: In the paragraph "Both apeglm and MLE..." the acknowledgement that comparing against CAT in a hold-out set is potentially problematic is a bit buried in the middle of the paragraph. It would seem better to acknowledge this up front. Given the problems with CAT acknowledged here I suggest removing that figure (Fig 3d) or moving to an Appendix.
- Figure 5: this should have a y axis that starts at 0.

Is the rationale for developing the new method (or application) clearly explained?

Yes

Is the description of the method technically sound?

Yes

Are the conclusions about the method and its performance adequately supported by the findings presented in the article?

Partly

If any results are presented, are all the source data underlying the results available to ensure full reproducibility?

Yes

Are sufficient details provided to allow replication of the method development and its use by others?

Partly

Reviewer Expertise:

Bayesian statistics; statistical genetics

I confirm that I have read this submission and believe that I have an appropriate level of expertise to confirm that it is of an acceptable scientific standard, however I have significant reservations, as outlined above.

## References

[1] : Flexible signal denoising via flexible empirical Bayes shrinkage. *arXiv*.2019; Reference source PMC1075102038149302

# Response

**Published:** 2020-11-01

**Authors:** Josh Zitovsky

**Affiliations:** University of North Carolina at Chapel Hill, USA

Summary:

 The paper presents new implementations of shrinkage methods for beta binomial models, implemented in the R software package apeglm. One potential application of these models is estimating allele-specific biases in various sequencing-based assays (and differences in bias between groups), and the paper focuses on this application.

 The performance of the shrinkage methods is assessed via simulation and real data analysis (using performance on hold-out data as a performance metric), and the shrinkage methods implemented here are found to be competitive with another shrinkage approach (adaptive shrinkage, ash), and consistently outperform the mle. The new implementations are also shown to be computationally faster than existing implementations (eg aod or the previous version of apeglm).

 The paper is generally well written, and carefully done, with some exceptions I note later. The new implementations seem likely to be useful in a range of applications. Certainly the use of shrinkage methods in these types of applications is to be encouraged, and I congratulate the authors for leading the way on this. I hope they will find my report helpful in revising their work.

 I was instructed "Please indicate clearly which points must be addressed to make the article scientifically sound." I believe points 2-4 below are most important to address to make the article scientifically sound.

*Thank you for your constructive comments and careful evaluation of our software and analysis. We found your report helpful and have tried our best to address all of your concerns. Point-by-point responses are provided below.*

**1. A note on differences between the shrinkage methods:**

 One thing that I felt was missing from the paper was a qualitative summary of how the two shrinkage methods used here differ from one another. Both are a form of Empirical Bayes shrinkage, but they use different prior families, different likelihoods, and different point estimate strategies: apeglm uses a Cauchy prior, with beta-binomial likelihood, and posterior mode point estimate; whereas ash uses a more flexible unimodal prior (which includes Cauchy as a special case), a normal approximation to the likelihood, and uses a posterior mean point estimate. So the trade-off here is that ash is using an approximate likelihood, but a more flexible prior and arguably a more principled point estimate (posterior mean is optimal under mean squared error).

 I think many readers might benefit from this "high-level" summary of the differences.

 Another important point, which will come up later, is that when using ash the user has a choice of how to make the normal approximation. Specifically ash requires the user to provide point estimates (beta-hat) and standard errors (s-hat), with the goal that beta-hat approx \sim N(beta, s-hat), where beta is true value that is being estimated.

 So there is not only one way to apply ash to a problem, but many different ways depending on the choice of point estimate beta-hat. The mle is one natural choice, but in this application there can be problems with infinite mles; see 2. Below.

*We agree that there are important methodological differences between the methods, and that  a high-level summary of these differences would be beneficial to the readers. We have added a paragraph highlighting these differences in the second-to-last paragraph of the “Estimation methods” subsection of the “Methods” section. Among other differences, we highlight the increased flexibility of ash’s prior and its ability to handle non-ML estimators. Additional details regarding the methodology of these methods have also been added to the sections where apeglm and ash were initially introduced.*

**2. On dealing with infinite mles:**

 To explain the issue with infinite mles, consider first a simple binomial experiment X \sim Bin(n,p) in which we observe X=0. Then the mle for p is 0, and the mle for theta:=log(p/(1-p)) is -Infinity. Similarly, if X=n the mle for theta is Infinity. Also, in both cases. the standard error for theta is infinite. The same issue arises in the more complex beta-binomial models considered here.

 Essentially if all the reads in an experiment show the same allele then the mle for the allelic bias parameter (on the logit scale) is +-Infinity. This could happen due to low coverage, but it could also happen at high coverage sites if the allelic bias is very strong.

 This issue appears to arise in the data analyses used to produce Figure 3 (I did not check whether it arises in the simulations). In Figure 3 there appear many mles (y axis) taking values near +-(5 to 6); however, my brief investigations of the data suggested that most of these likely correspond to genes where all the reads come from one allele, and so the mle is actually +-Infinity as above. (That these infinite mles are computed to be near +-6 is presumably due to an issue with the numerical maximization method used to compute the mle.)

 I suspect that the problems with ash observed in Fig 3 stem from this issue: the mle for these situations where all the reads come from one allele are very unstable, and have a very large standard error (technically infinite, although for numeric reasons finite values are used) and these large standard errors cause these mles to be shrunk excessively.

 A simple fix for this problem, and one I suggest the authors try, is to add a pseudo-count (say 1, or 0.5) to the counts for *each* allele in the data before computing "mles" and corresponding standard errors.

 Pseudo-counts are commonly used to improve stability of mles in this type of situation. Indeed, adding pseudo-counts can be viewed as a simple kind of shrinkage method, so it seems reasonable to compare the more sophisticated EB methods with the simple pseudo-count method. For most genes the point estimates and standard errors will be very little affected by the addition of a small pseudo-count; but for the problematic genes with infinite mle the pseudo-count will stabilize the point estimate and reduce the standard error. I suspect entering the stabilized estimates + standard errors into ash  will greatly reduce the problems observed with use of the mles in Figure 3.

 (Incidentally, Xing, Carbonetto and Stephens arXiv:1605.07787 1 encounter a closely-related issue when using ash to smooth Poisson data; they solved this using a slightly different approach that is conceptually similar to adding a pseudo-count.)

*As you suspected, there were indeed genes with “truly infinite” MLEs, but due to numerical reasons, were given finite estimates by the apeglm package. As you suggested, we have now performed additional analyses adding a pseudocount to each allele prior to computing MLEs, and compared the performance of the resulting ML, apeglm and ash estimates to those not involving pseudocounts for the simulations. We also attempted to remove the infinite ML genes prior to analysis. Results can be found in Table 1, Table 2 and Supplementary Figure 3.*

**3. Subsetting results based on shrinkage amounts and "true" values:**

 In several places the paper reports error measures on subsets of the results. For example, in Table 1 lines 2-4 involve subsets of results chosen based on the true effect size or shrinkage amount (which depends on the true effect). Although tempting, this type of result is hard to interpret. For example, even the optimal shrinkage rule (i.e. the one that uses the correct prior, likelihood and loss function) may not perform uniformly better than the mle on subsets that are chosen in this way. Thus the sentence on p7 ("For instance, among genes with effect sizes greater than two...") may also be true for the optimal shrinkage rule, and so does not constitute direct evidence for "overshrinkage". (I agree there is overshrinkage, but this is not the right way to show it). Comparisons like p9 ("Among genes that were shrunk..."), which stratify by the amount of shrinkage, have the same problem because the amount of shrinkage depends on the true value and not only on the observed value.

 It is much cleaner and easier to interpret results if they are subsetted based on the *observed* effect (mle), rather than the true effect. This is because the optimal shrinkage rule is still optimal for *any subset chosen based only on the observed data*. (For this reason you could also subset based on other features of the observed data, like total allele count.) For example, if a method is worse than the mle for the subset of results where the mle is >4 then this is indeed evidence of a problem of some kind.

*Shrinkage in the first manuscript was defined as the movement of apeglm and ash estimates from the MLE toward zero. As apeglm, ash and ML estimates are all functions of the observed data, the degree of shrinkage is also a function of observed data and thus we felt that subsetting by shrinkage was valid. However, we do agree with your concern that subsetting by true effect sizes may cause difficulty in contrasting procedures with each other with respect to the optimal shrinkage rule, and thus have removed results of mean absolute error stratified by the true effect sizes. Moreover, per your suggestion, we have added stratification of MAE by total gene counts and MLE magnitude. We also added MA plots, which illustrates how the amount of shrinkage differs by total gene counts and MLE size (these plots were previously in the Supplemental Material, but have been moved to the main paper).*

**4. Computation: speed vs accuracy:**

 When comparing with other methods/implementations there should be some assessment not only of speed, but of accuracy of the different implementations (meaning the accuracy with which they optimize the log-likelihood, rather than the accuracy of the point estimates). Fast answers are easy if you do not care about accuracy....

 E.g. I suggest boxplots of loglik(method) - loglik(apeglm-new) for each method, to show that the apeglm-new solution is consistently as high in log-likelihood as other methods (or nearly so). Are there convergence criteria decisions to be made that might affect the trade-off between speed and accuracy?

*We agree that an assessment of numerical accuracy is important in showcasing our package, and have adding such assessments in the new version of the manuscript. We focused our analysis of numerical accuracy on genes such that the difference in an estimated coefficient between apeglm and the other packages were non-negligible (above 0.01), and among those genes reported the differences in log-likelihood. A high-level overview of the results is present in the last paragraph of the “Computational Performance of Apeglm” subsection of the “Results” section, and a detailed summary of the results was added to the Supplementary Methods section. Overall, we found that our package is, in addition to its estimation speed, also numerically accurate.*

**5. Reproducibility:**

 I congratulate the authors on making all their code and data available. After a few tweaks to the code I was able to run the code used to produce Figures 1-3. However, my version of Fig 3 looked different from the one in the paper - my figure had different colors and some points seemed to be missing on my figure. I do not know the reasons for this.

 Reproducibility would have been made easier by avoiding the use of absolute file paths. I also suggest not defining functions that operate on global variables (e.g. subsetCalculations = function(sub){..,}) since they are more likely to lead to reproducibility problems.

 I was unable to run the code to perform the computation time comparisons (Figure 4), since it errored out. Again I do not know the reason, but it could be due to differences in the package versions I used compared with the authors. I did not have time to troubleshoot this.

*We apologize for the reproducibility issues in the first version of the paper. Briefly, the issues you reported stemmed from two underlying causes: 1) the version of the apeglm package in the devel branch at the time of publication did not match the version used in the manuscript; 2) we accidentally uploaded the wrong scripts to Zenodo. We have now correctly identified the apeglm package version in the manuscript (v1.11.2) and replaced the scripts in Zenodo with the correct ones. All scripts should now run without issues and output the same numbers and plots as shown in the paper. Moreover, we have removed absolute file paths and do not use global variables in our functions (some of the local variables defined within functions might share names with global variables created later on, but our functions no longer call global variables directly).*

**6. Miscellaneous other comments:**

 For Table 3, I think it should be noted that the coverage probability is expected to be <0.95 because you are looking at how often the interval covers the *estimate* in the larger dataset, and not the *true* value. This makes it a hard to compare the methods here because it isn't clear what the right coverage is.

*Due to concerns posed by yourself and other reviewers, we have completely rewritten our analysis of real data to focus on more qualitative results, and have mostly left evaluations of accuracy to the simulations, where the true simulation parameters are known. Among other changes, we do not evaluate or assess coverage probabilities of estimators when analyzing the real data.*

 p12: "ash would most likely perform best in a situation where most effects were small". I don't see any evidence for this here (e.g. in the normal simulation ash performs fine) and indeed no reason to expect it to be true a priori. I think this statement should be removed.

*We have removed this statement.*

**7. Minor comments:**

- p3: "When a subject is heterozygous for a gene at a particular SNP"; this wording seemed awkward to me.

*We have changed the wording to “When a subject is heterozygous at a particular SNP within an exon of a gene”*

- p3: "... making it the most robust and reliable when dealing with small sample sizes"; this conclusion ("making it") seemed not to follow directly from the first part of the sentence.

*We have changed this from “the most robust and reliable” to just “robust and reliable”.*

- p4: "Apeglm shrinks the effect of one predictor at a time": I think this sentence might work better at the start of the paragraph, before specifying the prior used.

*We have made the suggested change.*

- p5: "guided by the author's claim": this is not just a claim, it is a theorem dating back to the 1950s (see original paper for citations).

*Apologies for the confusion. We have changed it from “guided by the author’s claim” to “guided by the fact”  and have cited both ash and the original 1950’s citation.*

- p5: diallel typo?

*In our original manuscript, we had the term “diallel cross”, we did not find a typo.*

- p5: use of beta for the mean of the exponential distribution is confusing as beta is already used elsewhere.

*We changed the notation for the mean parameter from beta to mu.*

  

- p9: "We also conducted..." This did not seem worth reporting to me. The difference in sample size (5 vs 5 instead of 4 vs 4) is too small to expect that the results would be very different.

*We have removed this result.*

- p9: In the paragraph "Both apeglm and MLE..." the acknowledgement that comparing against CAT in a hold-out set is potentially problematic is a bit buried in the middle of the paragraph. It would seem better to acknowledge this up front. Given the problems with CAT acknowledged here I suggest removing that figure (Fig 3d) or moving to an Appendix.

*Please see our response to your concerns in point #6.*

  

- Figure 5: this should have a y axis that starts at 0.

*Unfortunately, the y-axis for figure 5 of the initial version of the paper (renamed Figure 8 in version 2) is on the log-scale, which means we cannot start it at zero. Using a log scale is necessary due to the very different computational times of the apeglm and aod packages and the difference in how well they scale with increasing numbers of covariates.  We considered changing the figure to start the y-axis at a smaller positive number (eg 10, 1, 0.1 etc.)  but we ultimately decided against this as the exact cut-point at which to start the y-axis would have been arbitrary and there would have been a large amount of unnecessary white space between the plots and the x-axis (due to the fact that the y-axis is measured on the log scale).*
